# Reconstructing severe acute respiratory infection dynamics from ICD-10 hospital discharge data: a 10-year analysis of 11.2 million discharges in Ecuador, 2014–2023

**DOI:** 10.3389/fpubh.2026.1863826

**Published:** 2026-07-22

**Authors:** Jaime Angamarca-Iguago, Jaen Cagua-Ordónez, Juan Marcos Parise-Vasco, Natasha Bella Fuentes-Tumbaco, Mónica Escobar-Naranjo, Claudia Reytor-González, Daniel Simancas-Racines

**Affiliations:** 1 Center for Evidence Ecosystems, Implementation Science and Decision-Making (CIDES), Facultad de Ciencias de la Salud y Bienestar Humano, Universidad Tecnológica Indoamérica, Ambato, Ecuador; 2 Laboratorio Clínico, Unidad de Apoyo Diagnóstico, Hospital Pablo Arturo Suárez, Quito, Ecuador; 3 Dirección Nacional de Vigilancia Epidemiológica, Ministerio de Salud Pública del Ecuador, Quito, Ecuador

**Keywords:** Ecuador, epidemiology, hospital discharges, ICD-10, respiratory tract infections, SARI, Serfling regression, surveillance

## Abstract

**Background:**

In many low- and middle-income countries, surveillance of severe acute respiratory infections (SARI) relies on administrative hospital data without virological confirmation, and its ability to capture epidemic dynamics and age-specific burden remains uncertain.

**Methods:**

We analyzed 11,232,698 hospital discharges recorded by Ecuador's National Institute of Statistics and Censuses between 2014 and 2023, identified SARI episodes using International Classification of Diseases, 10th Revision (ICD-10) codes under broad and length-of-stay–restricted case definitions, and incorporated a neonatal component that included perinatal respiratory codes for infants younger than 1 year. Serfling harmonic regression was used to estimate seasonal baselines and excess SARI admissions at national, provincial, and age-stratified levels.

**Results:**

Administrative data identified 538,272 SARI discharges and revealed marked heterogeneity in incidence and case fatality across provinces and age groups. A previously unrecognized shift in ICD-10 coding from respiratory to perinatal chapters produced an apparent 99% decline in infant SARI after 2014; reclassifying perinatal respiratory codes restored stable high SARI rates in infants and increased their estimated burden by nearly ten-fold. Nationally, we identified 50 epidemic weeks with 77,352 excess SARI discharges (95% CI 70,030–85,982), while the COVID-19 pandemic disrupted typical seasonality, reducing admissions but increasing mortality among older adults.

**Conclusions:**

Routinely collected hospital discharge data can reconstruct SARI epidemic dynamics and geographic disparities in the absence of virological surveillance, but are highly sensitive to coding practices. Incorporating neonatal perinatal respiratory codes into SARI definitions is essential to avoid underestimating infant burden and misinforming public health priorities.

## 1 Introduction

Severe acute respiratory infections (SARI) are a leading cause of hospitalization and mortality worldwide, particularly among children under 5 years and adults over 65 years (1, 2). In Latin America and the Caribbean, respiratory viruses, and respiratory syncytial virus (RSV) in particular, account for the largest share of hospital admissions for acute lower respiratory infection in children, with disease burden estimates ranging widely across the region and case fatality among hospitalized children of 0.5–2% in middle-income settings (3, 4). Adult SARI burden in the region remains less well characterized, but recent national-level studies, including a stratified mortality analysis of more than half a million SARI hospitalizations in Brazil, indicate that asthma and other chronic respiratory conditions remain important effect modifiers of in-hospital mortality (5). The World Health Organization recommends sentinel surveillance systems with virological confirmation for SARI monitoring (6); however, most low- and middle-income countries (LMICs) lack the laboratory infrastructure for systematic viral testing of hospitalized respiratory patients (7), and the few sentinel networks that exist in equatorial Latin America cover a small fraction of the hospital population and concentrate on a limited number of urban reference hospitals (4, 8).

An alternative approach uses administrative hospital discharge databases coded with the International Classification of Diseases, 10th Revision (ICD-10) to reconstruct respiratory disease dynamics at the population level (9, 10). This strategy has been applied successfully in high-income settings for estimating influenza-associated burden (11, 12) and has been formalized through methodological frameworks such as the Broad and Timely Case Definitions (BCD/TCD) proposed by Buda et al. (13) and the Serfling regression baseline model adapted by Rodrigues et al. (14). In Ecuador, the only previous comprehensive estimate of ARI burden, using mortality and morbidity records over 2011–2015, reported an annual loss of approximately 100,000–120,000 disability-adjusted life years driven by extreme-age mortality, and was limited to descriptive ICD-grouped aggregations without time-series modeling, virological linkage, or formal epidemic-excess estimation (15). To our knowledge, no Latin American study has applied a full Serfling-based excess-discharge framework to a decade-long national administrative dataset of this magnitude, nor evaluated the impact of intra-period ICD-10 coding shifts on age-specific SARI estimates.

Ecuador, a middle-income equatorial country of approximately 17 million inhabitants distributed across 283,561 km^2^, offers a particularly informative setting for such an analysis. The country spans four distinct ecoregions, the Pacific coast, the Andean highlands (with population centers at altitudes between 2,500 and 4,000 m), the Amazon basin, and the Galápagos archipelago, across which climate, healthcare access, and population age structure vary substantially. Its location on the equator produces a bimodal respiratory disease pattern associated with the transitions between the dry and rainy seasons rather than the single annual winter peak seen in temperate countries (8). Hospital care is delivered through a mixed public-private system and is captured uniformly by the National Institute of Statistics and Censuses (INEC), which maintains a comprehensive national hospital discharge registry covering every public and private discharge nationwide with ICD-10 diagnostic codes (16). This registry provides a unique opportunity to evaluate whether administrative data alone can characterize SARI epidemiology in an equatorial Latin American setting.

During preliminary analysis of this database, we observed a striking anomaly: SARI discharges in infants (< 1 year) dropped from over 10,000 annually in 2014 to fewer than 250 per year from 2015 onwards, despite stable overall infant hospitalization rates. Empirical re-extraction of the full 2007–2024 INEC microdata confirmed that this was caused by a shift in ICD-10 coding practices: neonatal respiratory conditions previously assigned to J-codes (J09–J22, acute lower respiratory infections) and to P-codes (P22, respiratory distress of newborn; P23, congenital pneumonia; P24, neonatal aspiration syndromes) in roughly equal proportions during 2007–2014 were almost exclusively assigned to P-codes from 2015 onward. This coding transition effectively rendered neonatal SARI invisible to surveillance systems relying solely on the standard J-code SARI definition, and is, to our knowledge, the first such transition documented for a Latin American national database.

The objectives of this study were: (1) to evaluate the feasibility of reconstructing SARI epidemic dynamics from administrative hospital discharge data without virological confirmation in an equatorial middle-income setting; (2) to quantify the impact of ICD-10 coding shifts on age-specific SARI burden estimates and to verify, with extended pre-period (2007–2013) and post-period (2024) data, that the observed change is administrative rather than epidemiological; (3) to estimate seasonal baselines and epidemic excess using Serfling harmonic regression at national and provincial levels, with explicit sensitivity to the 2014 coding-transition year; and (4) to characterize the epidemiological heterogeneity of SARI across Ecuador's 24 provinces over a 10-year period spanning pre-pandemic, pandemic, and post-pandemic phases, with both absolute and population-standardized metrics.

## 2 Methodology

### 2.1 Study design

We conducted a retrospective, population-based cross-sectional analysis of all hospital discharges registered in Ecuador from 1 January 2014 to 31 December 2023. The analytical approach comprised four sequential stages: (1) data extraction and quality control; (2) ICD-10 diagnostic phenotype mapping; (3) SARI case definition and weekly time-series construction; and (4) Serfling baseline estimation with epidemic excess quantification. The 10-year analytical window 2014–2023 was pre-specified at study inception. The 2024 INEC discharge data, which became available during revision, were used exclusively in the supplementary empirical verification of the ICD-10 coding shift (Methods 2.7, Supplementary Tables S1a and S1b) to test whether the 2014–2015 transition was permanent, and were not incorporated into the main Serfling, provincial, age-stratified, or composite SARI analyses to preserve the pre-specified analytical window.

### 2.2 Data source

Data were obtained from the “*Registros Estad*í*sticos de Camas y Egresos Hospitalarios*” maintained by Ecuador's Instituto Nacional de Estadística y Censos (INEC) (16). This administrative registry captures every hospital discharge (live discharges, deaths, and transfers) from all public and private health facilities nationwide. The dataset is released annually as anonymized, de-identified microdata files in CSV format through the INEC Open Data portal (https://www.ecuadorencifras.gob.ec/camas-y-egresos-hospitalarios-informacion-historica/). Ten annual files (2014–2023) were downloaded and concatenated into a single analytical dataset.

### 2.3 Variables extracted

The following variables were extracted from each discharge record (INEC variable names in parentheses) (Table 1).

**Table 1:** Operational definitions of variables extracted and derived from the national hospital discharge database.

Variable	Source field	Description
Demographic and spatial variables		
Sex	sexo	Male (1) or female (2)
Age (years)^*^	edad_years	Computed from primary age fields (continuous)
Age group (years)^*^	age_group	Categorized as < 1, 1–4, 5–19, 20–64, and ≥65
Ethnicity	etnia	Self-reported ethnicity code
Province of residence	prov_res	Patient's province of usual residence
Province of hospitalization	prov_ubi	Two-digit code for the province of the health facility (1–24)
Canton of hospitalization	cant_ubi	Canton code within the province
Temporal and admission variables		
Year of discharge	data_year	Administrative year of the record
Date of admission^†^	fecha_ingr	Date of hospital admission
Date of discharge^†^	fecha_egr	Date of hospital discharge
Length of stay (days)	dia_estad	Number of days from admission to discharge
Discharge condition	con_egrpa	Alive (1), dead (2), or dead-on arrival (3)
Clinical variables		
Principal diagnosis	cau_cie10	Full ICD-10 code of principal diagnosis at discharge
Diagnostic group^*^	causa3	First three characters of the principal diagnosis

Data are from the INEC hospital discharge records. ICD-10, International Classification of Diseases, 10th Revision.

^*^Derived variable not originally present as a single field in the raw dataset.

^†^Reconstructed from component fields (anio, mes, dia) when the original date string was missing or malformed.

### 2.4 Data quality and exclusions

Date fields were reconstructed from their year, month, and day component columns (‘anio_ingr‘, ‘mes_ingr‘, ‘dia_ingr‘) when the combined date string (‘fecha_ingr‘) was missing or unparseable. After date reconstruction and validation, all 11,232,698 discharge records had a valid admission date; therefore, no records were excluded for invalid admission dates (0 of 11,232,698; 0.00%). No records were excluded based on diagnosis, age, sex, or province. The total analytical sample comprised 11,232,698 discharge records.

### 2.5 ICD-10 diagnostic phenotype mapping

A structured diagnostic codebook was developed to map three-character ICD-10 codes to clinically meaningful diagnostic groups. Each entry in the codebook specifies: (1) the ICD-10 three-character prefix (e.g., J18); (2) a descriptive group name; (3) a machine-readable group code; (4) the literature reference or standard justifying inclusion; and (5) a justification string. The mapping was implemented as a vectorized prefix-match function: for each discharge record, the three-character prefix of the principal diagnosis (‘causa3‘) was matched against all codebook entries, and Boolean flags were generated for each diagnostic group.

The following diagnostic groups were defined:

- SARI (Severe Acute Respiratory Infection): J09–J22, following the Broad Case Definition (BCD) of Buda et al. (13). This range encompasses influenza (J09–J11), viral pneumonia (J12), bacterial and unspecified pneumonia (J13–J18), acute bronchitis (J20), acute bronchiolitis (J21), and unspecified acute lower respiratory infection (J22).
- SARI-NEO (Neonatal SARI): P22 (respiratory distress of newborn), P23 (congenital pneumonia), P24 (neonatal aspiration syndromes). These codes were added to correct a coding artifact identified during preliminary analysis.
- PI (Pneumonia and Influenza): J09–J18, following the P&I proxy of Rodrigues et al. (14). This narrower range excludes bronchitis and bronchiolitis codes (J20–J22).
- FLU (Influenza): J09–J11.
- COV (COVID-19): U07.1, following WHO emergency coding guidelines.
- Additional groups mapped for quality assurance included: Other ARI (J00–J06), Bronchitis/LRI (J20–J22), Viral Pneumonia (J12), Bacterial Pneumonia (J13–J18), Ischaemic Heart Disease (I20–I25, used as a non-respiratory control signal), Heart Failure (I50), and Lung Abscess (J85–J86). A single ICD-10 prefix can generate flags for multiple overlapping groups (e.g., J12 flags both SARI and PI).

### 2.6 Diagnostic and coding workflow in Ecuadorian hospitals

In Ecuador, the assignment of an ICD-10 diagnostic code to a hospital discharge is performed at the time of discharge by the attending physician, frequently with verification or correction by hospital coding staff before the record is transmitted to INEC. The underlying clinical workflow that informs the principal-diagnosis code is heterogeneous across both diagnostic categories and care settings, and we summarize it here because the heterogeneity is directly relevant to the interpretation of administrative SARI surveillance.

Pneumonia and unspecified lower respiratory infection (J18, J22): Diagnosis is made clinically, fever, cough, tachypnoea, hypoxaemia, and auscultatory findings, and is supported by chest radiography in the great majority of admissions to public and private hospitals. Microbiological confirmation is the exception rather than the rule outside intensive-care units and tertiary referral centers. The choice between J18 and J22 in adults is largely a coding habit and varies between hospitals.

Influenza (J09–J11): In principle, J10 (influenza with virus identified) and J09 (avian or pandemic influenza) require laboratory confirmation, while J11 (influenza, virus not identified) does not. In practice, J11 is the dominant influenza code in the INEC dataset because systematic virological confirmation is not routinely performed outside the SIVE-Alerta sentinel virology network (7), which is geographically concentrated in Quito and Guayaquil. The influenza signal we describe is therefore best interpreted as clinically-defined influenza rather than as laboratory-confirmed disease.

Bronchitis and bronchiolitis (J20, J21): Diagnosis is essentially clinical, with viral etiology rarely confirmed. J21 is overwhelmingly assigned to infants and young children and is supported by characteristic clinical features (wheezing, retractions, hypoxemia) without routine RSV testing outside academic units.

COVID-19 (U07.1, U07.2): From 2020 onwards, U07.1 (COVID-19 virus identified) was widely used in hospitals with PCR or rapid-antigen capacity, while U07.2 (COVID-19 virus not identified, clinical-epidemiological diagnosis) was applied where confirmatory testing was unavailable. Because U07.x codes are not in the J chapter, they are not captured by the standard BCD definition (J09–J22); we discuss this caveat in the Discussion.

Neonatal conditions (P22, P23, P24): Diagnosis is led by neonatologists at the time of newborn discharge. P22 (respiratory distress of newborn) is supported by clinical course, gestational age, and chest radiography; surfactant deficiency in premature infants is the dominant etiology and the condition is non-infectious. P23 (congenital pneumonia) requires clinical features of pneumonia in the early neonatal period and is occasionally supported by blood culture, tracheal aspirate culture, or maternal infection history. P24 (neonatal aspiration syndromes) is supported by amniotic fluid characteristics (typically meconium-stained), respiratory distress immediately after birth, and chest radiography; this condition is also non-infectious.

Standardization framework: Ecuador adopts the WHO ICD-10 manual (Spanish-language edition) without national addenda for the assignment of three-character codes. The Ministry of Public Health (MSP) maintains clinical practice guidelines for several conditions including community-acquired pneumonia, but these are diagnostic and management recommendations rather than coding mandates. As a result, the same clinical presentation may receive different ICD-10 codes across hospitals or across time, a feature that we exploit explicitly in point 2.7 to identify the systemic 2014–2015 coding shift in neonatal respiratory disease, and that we discuss in the Discussion as a structural limitation of administrative-data SARI surveillance.

### 2.7 Empirical verification of the ICD-10 coding shift

Because case definitions based on respiratory ICD-10 codes are sensitive to coding behavior, we verified empirically that the abrupt change in infant J-code usage observed at the start of our study period reflected a systemic shift in coding practice rather than a localized registration artifact. To do this, we extended our analysis backward by seven years (2007–2013) and forward by 1 year (2024) using the same INEC Egresos Hospitalarios microdata, yielding an enlarged dataset of 19,859,700 discharge records covering 2007–2024. All discharges with a three-character ICD-10 prefix in J09–J22 (respiratory chapter) or P22–P24 (perinatal chapter) and a recorded age of less than 1 year were extracted, and a P-share statistic was computed for each year and each of the 24 provinces as P / (J + P).

Three observations supported the systemic nature of the shift: (1) During 2007–2014, J-codes and P-codes coexisted in infant respiratory hospitalizations with a national P-share ranging between 0.384 and 0.547; the change from 2013 (P-share = 0.480) to 2014 (0.547) was within the historical range. Between 2014 (10,606 J-coded discharges nationwide) and 2015 (197 J-coded discharges), J-coding for infant respiratory disease collapsed by 98.1%, while P-coded discharges (P22–P24) remained essentially stable (12,821 in 2014; 10,725 in 2015).

(2) The shift was geographically synchronous: 23 of 24 provinces (95.8%) showed a reduction of ≥90% in J-coded infant discharges from 2014 to 2015 (the only exception was the small-population Galápagos archipelago). The 2014 → 2015 national P-share jump of +0.435 is the largest year-to-year change observed in the 18-year series.

(3) The shift was not permanent: in 2024, J-coding partially returned (7,580 J-coded vs 3,013 P-coded infant discharges, P-share = 0.284). Although outside the study period, this reversal reinforces the interpretation that the 2014–2015 transition was administrative rather than epidemiological and is consistent with literature showing that ICD-10 coding behavior for respiratory conditions remains dynamic over time (17, 18).

### 2.8 Composite SARI case definition

The standard SARI BCD definition (J09–J22) was applied as the baseline. During preliminary analysis, we observed that SARI counts in infants (< 1 year) dropped from 10,606 in 2014 to 197 in 2015 (a 98.1% decline; see 2.7 for empirical verification across 2007–2024), while total infant discharges remained stable at 42,000–48,000 per year. Investigation of the full ICD-10 code distribution revealed that, from 2015 onwards, the vast majority of neonatal respiratory conditions were coded under Chapter XVI of ICD-10 (Certain conditions originating in the perinatal period: P22, P23, P24) rather than Chapter X (Diseases of the respiratory system: J09–J22). This transition was consistent across all provinces and was not accompanied by any documented change in clinical practice or disease incidence.

To correct this artifact, we defined a composite SARI indicator as follows:

- For age groups ≥1 year is BCD = is SARI (i.e., J09–J22 only).
- For age group < 1 year is BCD = is SARI OR is SARI-NEO (i.e., J09–J22 OR P22–P24).

Because the perinatal-chapter codes included in the composite definition span both infectious (P23, congenital pneumonia) and non-infectious (P22, respiratory distress of the newborn; P24, neonatal aspiration syndromes) conditions, we also report a more restrictive sensitivity definition (J09–J22 + P23 only). This restricted definition is intended for applications where only the infectious component of the infant burden is relevant, most importantly, the cost-effectiveness modeling of RSV-prevention interventions.

### 2.9 SARI case definitions

Following Buda et al. (13), two case definitions were operationalized:

- Broad Case Definition (BCD): Principal discharge diagnosis matching the composite SARI indicator (J09–J22 for all ages; J09–J22 or P22–P24 for < 1 year). This is analogous to the original BCD, which identifies SARI from billing codes alone.
- Timely Case Definition (TCD): BCD with length of stay (‘dia_estad‘) ≤ 7 days. In the original Buda et al. framework, TCD additionally requires a secondary diagnosis of a specific respiratory condition; since secondary diagnoses are not available in the INEC database, we adapted TCD as a length-of-stay filter, which serves as a proxy for community-acquired rather than nosocomial or chronic respiratory disease.

### 2.10 Epidemiological time series

Each discharge was assigned to an ISO 8601 epidemiological week and year based on its admission date (‘fecha_ingr‘) using the ‘isocalendar‘ method in Python's ‘pandas‘ library. Weekly time series were constructed at three levels of aggregation:

1. National: Total discharges, BCD SARI count, TCD-SARI count, BCD deaths, BCD proportion, and BCD case fatality ratio per epidemiological week.
2. Provincial: BCD and TCD counts per province (24 provinces, INEC codes 1–24) per epidemiological week.
3. Age-stratified: BCD and TCD counts per age group (< 1, 1–4, 5–19, 20–64, ≥65 years) per epidemiological week.
4. Province × age: Cross-classified counts for provincial age-specific analyses.

### 2.11 Serfling baseline and epidemic excess estimation

We estimated seasonal baselines and quantified epidemic excess following the Serfling harmonic regression framework (19), as adapted by Rodrigues and colleagues (14) for hospital admission data. The model assumes that non-epidemic respiratory admissions follow a predictable seasonal pattern described by:


$$\begin{array}{l} E\left[Y_{t}\right]=\beta_{0}+\beta_{1}t+\overset{K}{\underset{k=1}{\sum }}\left[\alpha_{k}\sin\left(\frac{2\pi kt}{52.18}\right)+\gamma_{k}\cos\left(\frac{2\pi kt}{52.18}\right)\right] \end{array}$$

Where Y_t_ is the observed weekly discharge count at sequential week t; β0 is the intercept (baseline level); β1 is the linear trend coefficient; α_*k*_ and γ_*k*_ are the sine and cosine coefficients for the k-th harmonic; K = 2 harmonics (annual period at 52·18 weeks and semi-annual at 26·09 weeks); and 52·18 is the average number of ISO weeks per Gregorian year (365·25/7).

The model was fitted using Ordinary Least Squares (OLS). An iterative exclusion procedure was employed: (1) the model was first fitted to all available weeks; (2) weeks where the observed count exceeded the fitted value plus 1.645 standard deviations of the residuals (one-sided 95th percentile) were classified as epidemic weeks and excluded from the fitting set; (3) the model was refitted on the remaining non-epidemic weeks; and (4) steps ii–iii were repeated for three iterations until the set of excluded weeks stabilized. This iterative approach ensures that epidemic peaks do not inflate the baseline estimate (11, 14).

The epidemic threshold was defined as the fitted baseline plus 1.645 times the standard deviation of the non-epidemic residuals, corresponding to a one-sided 95% prediction interval (19).

Epidemic excess for each week was calculated as:


$$\begin{array}{l} Excess_{t}=\max\left(Y_{t}-Ê\left[Y_{t}\right],\;0\right) \end{array}$$

Cumulative excess was computed by summing weekly excess values over the study period. Serfling baselines were fitted independently at (a) the national level for both BCD-SARI and P&I time series, and (b) for each of 24 provinces for BCD-SARI.

### 2.12 Severity indicators

In-hospital case fatality ratio (CFR) was calculated as:


$$\begin{array}{l} CFR=\frac{Discharges\;resulting\;in\;death}{Total\;SARI\;discharges}\times 100 \end{array}$$

Where the numerator includes all cases where the discharge condition was recorded as either dead during hospitalization (code 2) or dead-on arrival (code 3). CFR was computed nationally, by age group, by province, and by year.

Length of stay (LOS) was reported as mean and median days for SARI cases, stratified by age group and province.

Provincial rates were computed using INEC's 2018 population projections (midpoint of the study period) as the denominator (20). We report two per-100,000 metrics in Supplementary Table S4: the average annual SARI hospitalization rate (total BCD across 10 years divided by population × 100,000 per year) and the cumulative excess per 100,000 inhabitants (provincial Serfling excess over 10 years divided by population × 100,000).

### 2.13 Age group classification

Patients were classified into five age groups based on age in completed years:

This classification follows WHO recommendations for respiratory disease surveillance age stratification (21) and is consistent with Buda et al. (13) (Table 2).

**Table 2:** Age group classifications and rationales for severe acute respiratory infection surveillance.

Age group (years)	Definition	Rationale
< 1	0 to < 1	Neonatal and infant: affected by the ICD-10 coding shift
1–4	1 to 4	Preschool: the highest absolute SARI admission burden
5–19	5 to 19	School-age through adolescence
20–64	20 to 64	Working-age adults
≥65	≥65	Older adults: highest CFR

CFR, case fatality ratio; ICD-10, International Classification of Diseases, 10th Revision; SARI, severe acute respiratory infection; WHO, World Health Organization.

### 2.14 Software and reproducibility

All analyses were performed using Python 3.13.2. Key libraries and versions: pandas 2.2.3, NumPy 2.2.1, SciPy 1.15.1, statsmodels 0.14.4, matplotlib 3.10.0, seaborn 0.13.2. The analytical pipeline consists of modular scripts executed sequentially, with intermediate outputs saved as Apache Parquet files for efficiency and reproducibility.

### 2.15 Ethical considerations

This study used secondary, anonymized and publicly accessible data from the INEC open data catalog. Individual patient identifiers (name, national identification number, address) are not included in the INEC Egresos Hospitalarios microdata files, which guarantees the confidentiality of the information. Because no individual-level data was collected, no specific ethics approval was required. These considerations ensure that the analysis is consistent with the ethical standards applicable to research based on population data. The data are released under INEC's open data policy for research, educational, and policy use. This study is exempt from prior Comité de Ética de Investigación en Seres Humanos (CEISH) review under Section 1 of Acuerdo Ministerial 0075–2017 of the Ministry of Public Health, which classifies retrospective analyses of anonymised registry data as not requiring prior ethics-committee approval.

The study was conducted in accordance with the principles of the Declaration of Helsinki (2013 revision) for the ethical use of health data, and is consistent with the CIOMS International Ethical Guidelines for Health-related Research Involving Humans (2016) provisions on the use of stored data and biological materials, in particular Guideline 12, which permits secondary use of fully anonymized public data without individual consent provided that institutional oversight has confirmed the anonymization status.

## 3 Results

### 3.1 Study population

From 1 January 2014 to 31 December 2023, the INEC database contained 11,232,698 hospital discharges from 24 provinces. Annual discharge volume ranged from 907,515 in 2020 (reflecting pandemic-related reductions in elective hospitalizations) to 1,170,813 in 2023. Males accounted for approximately 46% of discharges. The age distribution of total discharges was: < 1 year: 499,747 (4.4%); 1–4 years: 658,225 (5.9%); 5–19 years: 1,783,912 (15.9%); 20–64 years: 6,609,799 (58.8%); ≥65 years: 1,681,015 (15.0%).

### 3.2 Overall SARI burden

Using the composite BCD definition, 538,272 discharges met the SARI case definition (4.79% of all hospitalizations). Of these, 417,663 also met the TCD definition (3.72%), indicating that 77.6% of SARI patients had a length of stay ≤ 7 days. The P&I subset (J09–J18) totalled 369,744 (3.29%), representing 68.7% of all SARI discharges. There were 21,406 in-hospital deaths among SARI patients, yielding an overall CFR of 3.98%.

**Figure 1:**
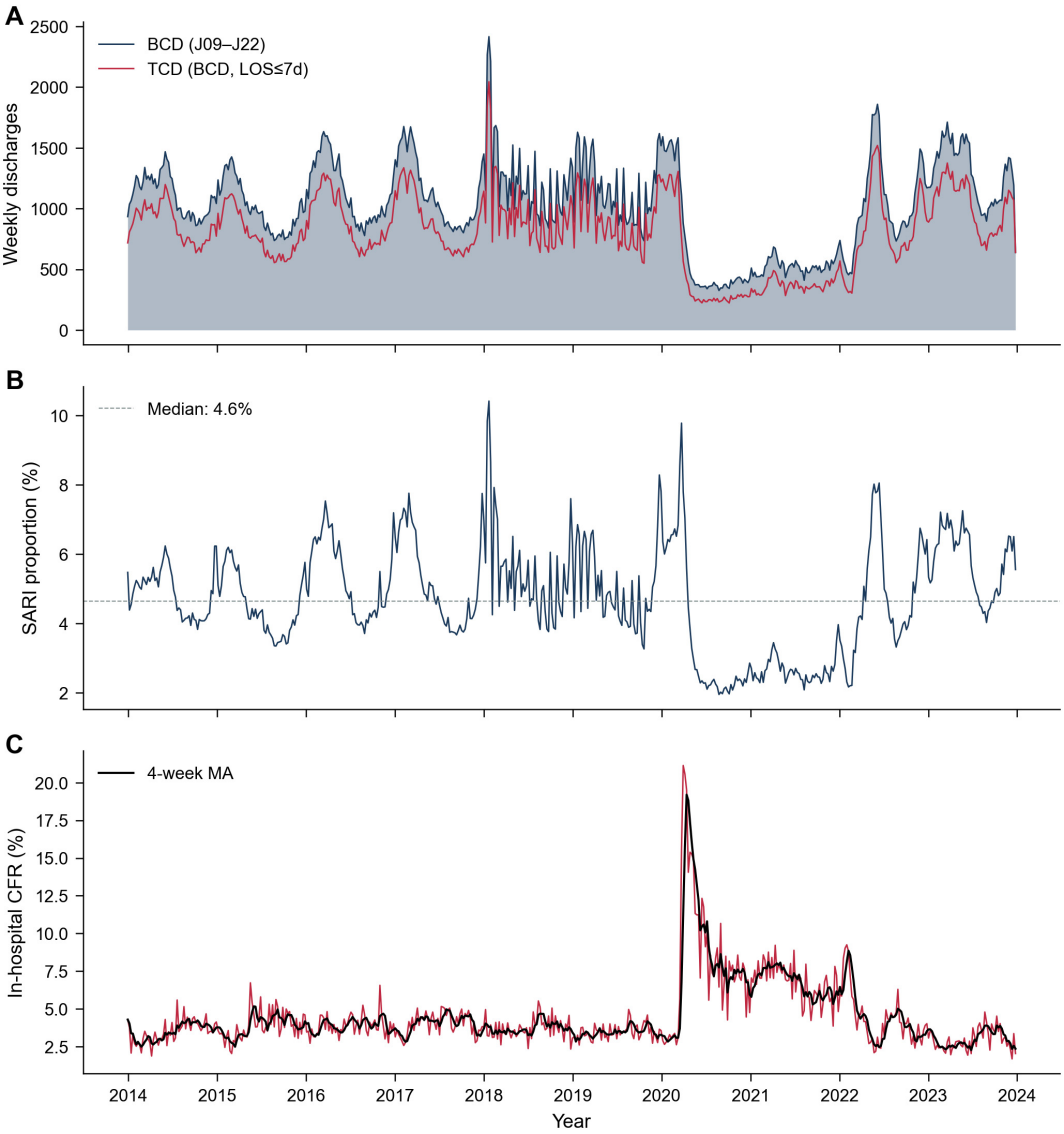
Temporal trends of severe acute respiratory infection hospitalizations and mortality, 2014–2023. **(A)** Weekly absolute number of hospital discharges meeting the Broad and Timely Case Definitions. **(B)** Proportion of SARI discharges relative to all-cause hospitalizations, with the dashed line representing the historical median (4·6%). **(C)** In-hospital case fatality ratio among SARI patients, smoothed with a 4-week moving average. BCD, Broad Case Definition; CFR, case fatality ratio; LOS, length of stay; MA, moving average; P&I, pneumonia and influenza; SARI, severe acute respiratory infection; TCD, Timely Case Definition.

Annual SARI volume showed a clear pattern (Figure 1; Table 3): an initial period of relatively stable counts (52,563–64,063 per year during 2015–2018), followed by a pandemic disruption (35,918 in 2020 and 27,131 in 2021), and a post-pandemic rebound reaching the highest recorded level of 68,212 in 2023–151% increase from the 2021 nadir and 7% above the pre-pandemic 2018 peak.

**Table 3:** Hospital discharges for severe acute respiratory infection by year and age group in Ecuador, 2014–23.

Year	< 1 year	1–4 years	5–19 years	20–64 years	≥65 years	Total
2014	23,427^*^	14,967	4,385	5,561	9,642	57,982
2015	10,290	17,764	9,705	5,019	9,785	52,563
2016	10,170	20,935	11,776	6,377	10,726	59,984
2017	10,199	19,890	11,717	5,676	11,473	58,955
2018	9,680	21,121	12,807	7,791	12,664	64,063
2019	8,845	20,696	11,942	6,225	12,191	59,899
2020	7,755	8,926	5,208	6,418	7,611	35,918
2021	9,330	5,856	3,317	3,439	5,189	27,131
2022	9,892	19,379	10,646	4,364	9,284	53,565
2023	9,575	26,311	15,815	5,519	10,992	68,212

The data represent discharges meeting the Broad Case Definition. BCD-SARI, Broad Case Definition of severe acute respiratory infection; ICD-10, International Classification of Diseases, 10th Revision.

^*^The 2014 infant count is inflated by dual J and P coding (ICD-10) during the transition year to the new coding system.

### 3.3 Age-specific SARI burden

Children aged 1–4 years contributed the largest absolute number of SARI discharges (175,845; 32.7%), followed by infants < 1 year (109,163; 20.3%), adults ≥65 years (99,557; 18.5%), children 5–19 years (97,318; 18.1%), and working-age adults 20–64 years (56,389; 10.5%). However, the relative frequency of SARI within each age group's total discharges varied markedly: SARI accounted for 26.72% of all discharges in children 1–4 years, 21.84% in infants < 1 year, 5.92% in adults ≥65 years, 5.46% in children 5–19 years, and only 0.85% in working-age adults (Table 4).

**Table 4:** Burden of severe acute respiratory infection by age group using classic vs. composite definitions in Ecuador, 2014–23.

Age group (years)	Total discharges	Classic definition (J09–J22)	Neonatal codes (P22–P24)	Composite definition	Classic definition (%)	Composite definition (%)	Relative change (%)
< 1	499,747	10,990	98,173	109,163	2.20	21·84	+893.3
1–4	658,225	175,845	7,480^*^	175,845	26.72	26·72	0.0
5–19	1,783,912	97,318	3,083^*^	97,318	5.46	5·46	0.0
20–64	6,609,799	56,389	0	56,389	0.85	0·85	0.0
≥65	1,681,015	99,557	0	99,557	5.92	5·92	0.0

ICD-10, International Classification of Diseases, 10th Revision. ^*^ICD-10 P-codes (P22–P24) in the 1–4 and 5–19 age groups were not included in the composite definition (which was applied exclusively to the < 1 year group); these counts are shown for reference only.

### 3.4 Impact of the ICD-10 coding shift on infant SARI

The most striking finding was the near-complete disappearance of infant SARI when the standard BCD definition was used. In 2014, 10,606 infants were discharged with SARI J-codes (J09–J22), yielding a rate of 1,281 per 10,000 infant discharges. In 2015, this dropped to 65 cases (rate: 13.6 per 10,000) a 99.4% reduction. For the period 2015–2023, classic infant SARI averaged only 43 cases per year (range: 19–66), despite a stable total infant discharge volume of 42,000–48,000 per year.

Concurrently, 98,173 infant discharges during 2014–2023 carried P-codes: P22 (respiratory distress of newborn), P23 (congenital pneumonia), or P24 (neonatal aspiration syndromes). The annual volume of P-coded infant respiratory discharges was remarkably stable: 7,732–10,225 per year from 2015–2023 (coefficient of variation: 9.2%), a pattern consistent with a true underlying disease burden rather than a variable artifact.

The composite definition (J09–J22 plus P22–P24 for < 1) restored infant SARI to plausible levels (Table 5).

**Table 5:** Infant (< 1 year) severe acute respiratory infection using classic vs. composite definitions by year.

Year	Total infant discharges	Classic definition (J-codes)	Neonatal definition (P-codes)	Composite definition	Rate (per 10,000)	Classic definition CFR (%)	Composite definition CFR (%)
2014	82,792	10,606	12,821	23,427^*^	2,829.6	0.87	2.23
2015	47,968	65	10,225	10,290	2,145.2	0.00	3.95
2016	47,530	52	10,118	10,170	2,139.7	0.00	4.19
2017	48,355	66	10,133	10,199	2,109.2	0.00	5.49
2018	47,937	37	9,643	9,680	2,019.3	0.00	5.79
2019	48,209	46	8,799	8,845	1,834.7	2.17	4.12
2020	41,950	23	7,732	7,755	1,848.6	17.39	5.08
2021	43,377	19	9,311	9,330	2,150.9	0.00	5.48
2022	46,010	35	9,857	9,892	2,150.0	0.00	5.30
2023	45,619	41	9,534	9,575	2,098.9	2.44	3.95

CFR, case fatality ratio; ICD-10, International Classification of Diseases, 10th Revision; SARI, severe acute respiratory infection.

^*^The 2014 count is artificially inflated due to simultaneous J-code and P-code assignment during the transition year to the new coding system.

Three key observations emerged from this analysis. First, the composite rate was stable across the 2015–2023 period (range: 1,834.7–2,150.9 per 10,000; mean: 2,066.3; CV: 5.6%), strongly suggesting that the true burden remained constant and the coding shift was purely administrative. Second, classic CFR was unreliable, the tiny denominator of J-coded infants (19–66 per year) produced unstable CFR estimates, including 0.0% in years with zero deaths among J-coded cases and an implausible 17.39% in 2020 (reflecting 4 deaths among only 23 cases). In contrast, composite CFR was stable (3.95–5.79%) and clinically plausible for hospitalized neonatal respiratory disease. Third, the 2014 composite count (23,427) was approximately double the 2015–2023 average (9,526), consistent with dual coding during the transition year when both J-codes and P-codes were assigned to the same clinical conditions.

### 3.5 Severity by age group

**Figure 2:**
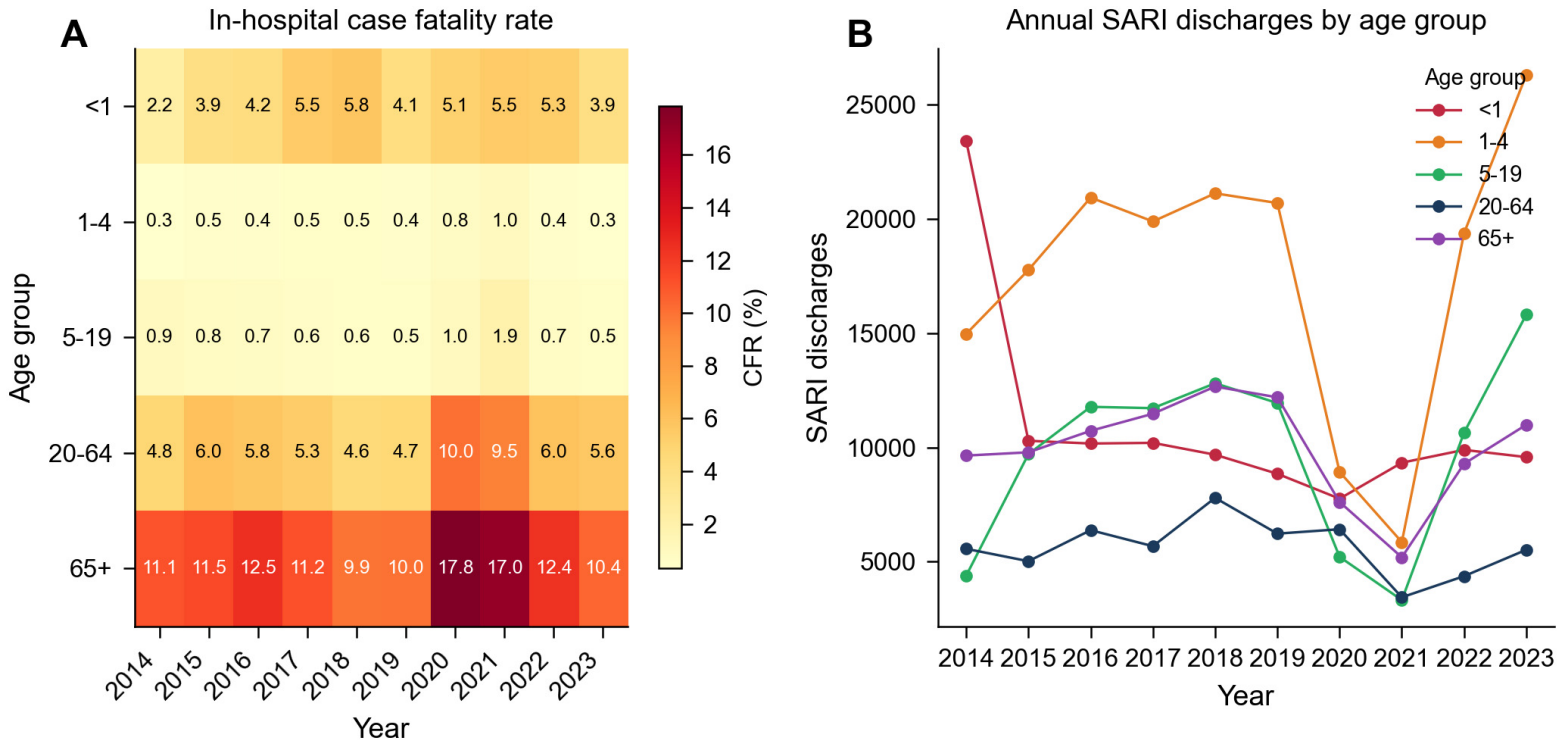
Severe acute respiratory infection severity and discharge volume by age group, 2014–2023. **(A)** Heatmap of in-hospital case fatality ratio by age group and year. **(B)** Annual absolute number of SARI discharges by age group using the composite definition. CFR, case fatality ratio; SARI, severe acute respiratory infection.

Case fatality displayed a U-shaped (bathtub-shaped) pattern by age, with the lowest mortality among preschool and school-age children and the highest at the extremes of age (Figure 2, Table 6). The lowest CFR was observed in children 1–4 years (0.46%), rising modestly in school-age children (5–19 years: 0.70%), then increasing sharply to 4.26% in infants < 1 year, 6.08% in working-age adults (20–64 years), and 11.89% in older adults (≥65 years), a 26-fold gradient from the lowest to highest age-specific CFR. Males predominated in pediatric SARI admissions (54–58%) but not in adult or older-adult SARI (47–50%). Length of stay was longest in infants (mean 8.8 days), reflecting the complexity of neonatal respiratory care that includes a substantial fraction of P-coded preterm and aspiration syndromes, and shortest in children 1–4 years (mean 5.0 days).

**Table 6:** Severe acute respiratory infection severity indicators by age group using the composite definition in Ecuador, 2014–23.

Age group (years)	SARI discharges	Deaths	CFR (%)	Mean LOS (days)	Median LOS (days)	Mean age (years)	Male (%)
< 1	109,163	4,646	4.26	8.8	5.0	0.1	57.5
1–4	175,845	811	0.46	5.0	4.0	2.1	54.1
5–19	97,318	682	0.70	5.2	4.0	8.1	54.6
20–64	56,389	3,431	6.08	7.7	4.0	45.8	49.9
≥65	99,557	11,836	11.89	7.3	5.0	81.1	46.6

CFR, case fatality ratio; LOS, length of stay; SARI, severe acute respiratory infection.

### 3.6 Temporal trends in SARI among older adults

Older adults (≥65 years) showed distinctive temporal patterns warranting detailed analysis (Table 7).

**Table 7:** Severe acute respiratory infection in older adults (≥65 years) by year in Ecuador, 2014–23.

Year	Total discharges (≥65 years)	SARI discharges	Rate (per 10,000)	Deaths	CFR (%)	Mean LOS (days)
2014	155,484	9,642	620.1	1,071	11.11	6.7
2015	156,707	9,785	624.4	1,121	11.46	6.9
2016	155,607	10,726	689.3	1,342	12.51	6.4
2017	163,955	11,473	699.8	1,283	11.18	6.4
2018	172,806	12,664	732.8	1,256	9.92	6.4
2019	180,621	12,191	674.9	1,223	10.03	6.3
2020	138,428	7,611	549.8	1,358	17.84	6.7
2021	169,531	5,189	306.1	884	17.04	6.2
2022	189,707	9,284	489.4	1,150	12.39	5.9
2023	198,169	10,992	554.7	1,148	10.44	5.9

CFR, case fatality ratio; LOS, length of stay; SARI, severe acute respiratory infection.

During the pre-pandemic period (2014–2019), SARI CFR among older adults ranged between 9.92% and 12.51%. The COVID-19 pandemic caused two simultaneous effects: (i) a reduction in SARI volume (from 12,191 in 2019 to 7,611 in 2020 and 5,189 in 2021), reflecting decreased circulation of typical respiratory pathogens due to non-pharmaceutical interventions, and (ii) a sharp increase in case fatality to 17.84% in 2020 and 17.04% in 2021, representing a 64–78% increase above the pre-pandemic average (10.87%). This divergence (fewer cases but more deaths per case) is consistent with the disproportionate severity of COVID-19 in older adults. By 2023, both SARI volume (10,992) and CFR (10.44%) had returned near pre-pandemic levels.

### 3.7 Serfling baseline and epidemic excess

#### 3.7.1 National level

**Figure 3:**
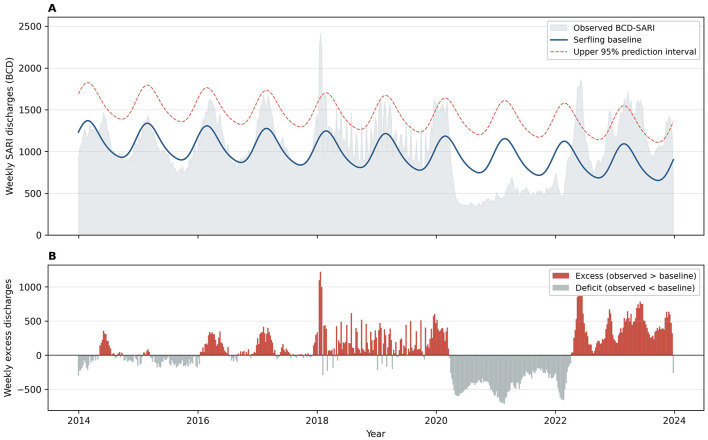
Serfling harmonic regression baseline and epidemic excess for severe acute respiratory infection, 2014–2023. **(A)** Observed weekly SARI discharges (Broad Case Definition) plotted against the fitted Serfling baseline and the upper 95% prediction interval (epidemic threshold). **(B)** Weekly excess discharges are calculated as the difference between observed counts and the expected baseline during epidemic periods. Deficits (observed counts below the baseline) are shown in gray. BCD, Broad Case Definition; PI, prediction interval; SARI, severe acute respiratory infection.

The Serfling harmonic regression model was fitted to 522 epidemiological weeks of national data covering 2014–2023 (Figure 3). Sensitivity analyses under alternative fitting strategies are reported in Supplementary Figure S2 and Supplementary Table S2. For BCD-SARI, the model identified 50 epidemic weeks (9.6%), with a cumulative excess of 77,352 (95% prediction interval 70,030–85,982) discharges above the seasonal baseline. For pneumonia and influenza, 51 epidemic weeks (6.9%) exceeded the threshold, with a cumulative excess of 110,809 discharges.

Epidemic weeks were concentrated in specific temporal waves corresponding to annual respiratory disease seasons. The pre-pandemic period (2014–19) showed regular annual peaks, with the most intense epidemic activity occurring in early calendar weeks (epidemiological weeks 1–12), consistent with a tropically modulated respiratory season. The pandemic period (2020–21) was characterized by suppressed epidemic activity, with fewer weeks exceeding the Serfling threshold despite increased case fatality. The post-pandemic rebound (2022–23) showed intense and sustained epidemic activity; although the highest single-week excess occurred during the 2018 peak, the post-pandemic seasons exhibited notably broader epidemic waves, resulting in a massive cumulative excess.

#### 3.7.2 Provincial level

Serfling baseline analysis was performed independently for each of the 24 provinces (Table 8). Provincial baselines revealed substantial heterogeneity in both epidemic intensity and timing. Two contrasting provincial archetypes were identified:

**Table 8:** Severe acute respiratory infection hospitalization metrics, epidemic activity and case fatality ratio in selected provinces of Ecuador, 2014–2023 (Broad Case Definition).

Province	Total discharges	Mean weekly discharges	Peak weekly discharges	Epidemic weeks	Epidemic weeks (%)	Total excess	Deaths	CFR (%)
Pichincha	119,765	229.4	748	97	18.6	29,782	3,733	3.12
Guayas	111,877	214.3	518	82	15.7	15,366	6,221	5.56
Azuay	38,664	74.1	178	80	15.3	7,168	1,894	4.90
Manabí	37,296	71.4	158	74	14.2	6,576	1,477	3.96
El Oro	28,361	54.3	126	61	11.7	4,500	1,437	5.07
Tungurahua	20,593	39.5	127	100	19.2	5,630	837	4.06
Morona Santiago	15,103	28.9	68	98	18.8	3,054	282	1.87
Galápagos	581	1.1	7	90	17.2	326	16	2.75

The data represent discharges meeting the Broad Case Definition. Selected provinces are shown here for contrast. CFR, case fatality ratio.

High-baseline, high-mortality archetype (Guayas): This province recorded the highest mean weekly count among reporting weeks (198.3 discharges per week) and the highest CFR (5.55%) of any province, yet only 6.2% of its weeks were classified as epidemic. Unlike Pichincha, which recorded the highest absolute peak (748 discharges per week), the pattern in Guayas suggests a sustained, high-baseline burden with relatively modest seasonal fluctuation. This is consistent with the hot, humid coastal climate of Guayas and potential continuous healthcare system saturation in the Guayaquil metropolitan area.

High-rate, low-mortality archetype (Morona Santiago): This province recorded a high proportion of epidemic weeks (18.8%) and the highest SARI rate per 10,000 discharges (983.3), yet it had the lowest CFR of any province (1.87%). This suggests a lower threshold for SARI hospitalization in this Amazonian province, possibly reflecting limited outpatient management capacity, a younger demographic profile, or more proactive admission criteria.

### 3.8 Provincial heterogeneity in SARI rates and case fatality

Severe acute respiratory infection rates per 10,000 discharges varied 2.9-fold across provinces, ranging from 341.6 in Galápagos to 983.3 in Morona Santiago. The five provinces with the highest rates were Morona Santiago (983.3), Cañar (715.0), Carchi (660.8), Zamora Chinchipe (569.2), and Loja (545.4). All of these are located in the Andean highlands or the Amazon region, suggesting that geographic and climatic factors, access to care, or population demographics might contribute to the observed differences.

Case fatality ranged 3.0-fold, from 1.87% (Morona Santiago) to 5.55% (Guayas). There was a notable inverse ecological correlation between the SARI admission rate and the CFR: provinces with higher SARI admission rates tended to have lower CFRs, and vice versa. The three provinces with a CFR >5% were Guayas (5.56%), Esmeraldas (5.16%) and El Oro (5.07%), predominantly coastal provinces with larger urban hospital systems. This pattern is consistent with the hypothesis that high-rate provinces might hospitalize milder cases (a lower admission threshold), whereas low-rate provinces hospitalize predominantly severe cases (a higher admission threshold), resulting in a higher observed CFR.

### 3.9 Provincial concentration of excess burden

In absolute terms, Pichincha and Guayas together accounted for 231,642 of 537,638 BCD-SARI discharges (43.1%) and 45,148 of 108,822 provincial-level excess discharges (41.5%) over the 2014–2023 period. However, this apparent concentration largely mirrors their share of the national population (7.40 million of 17.04 million inhabitants in 2018, 43.4%), and disappears after population standardization. The Pichincha + Guayas combined annual SARI rate is 313.0 per 100,000 inhabitants per year, against 317.3 in the other 22 provinces combined and a national average of 315.4. The cumulative Serfling excess per 100,000 inhabitants (10-year total) is 610.1 in Pichincha + Guayas, against 660.2 in the other 22 provinces (a ratio of 0.92 × ), and 638.4 nationally. After adjustment for population, the metropolitan provinces therefore do not show a disproportionate per-capita burden.

The genuine geographic outliers, when measured per capita, are small-population Amazonian and Andean provinces (Supplementary Table S4): Morona Santiago (3,054 cumulative excess discharges, 1,542.7 per 100,000), Pastaza (1,154; 1,089.1), Zamora Chinchipe (1,237; 1,076.1), Galápagos (326; 1,019.9) and Cañar (2,810; 1,003.9). These five provinces, despite contributing fewer than 9,000 cases in absolute terms, sustain the highest population-standardized excess. Guayas, the largest absolute contributor, has the lowest per-capita excess (353.3 per 100,000) among the most populous provinces, consistent with its high baseline burden and high CFR archetype: weekly hospitalisations are persistently elevated rather than punctuated by sharp epidemic peaks, so cumulative excess above the seasonal baseline is modest even though absolute volume is highest.

### 3.10 Length-of-stay filter (TCD): BCD–TCD concordance and LOS sensitivity

Of the 537,638 BCD-SARI discharges identified over the clean 522-week 2014–2023 window, 417,489 (77.6%) had a length of stay ≤ 7 days and therefore met the TCD definition. The BCD/TCD ratio varied systematically by age: 86.9% in 1–4 years, 85.6% in 5–19 years, 74.1% in 20–64 years, 68.5% in ≥65 years, and 65.7% in infants < 1 year. The lower TCD share at the extremes of age reflects the longer mean hospitalization observed in infants (mean LOS 8.8 days, driven by neonatal P-coded conditions with surfactant deficiency and complex aspiration syndromes) and in older adults (mean LOS 7.3 days, driven by comorbidity-related complications). In contrast, pediatric cases between 1 and 19 years recovered in < 7 days in most instances, consistent with the typical course of community-acquired lower respiratory infections at those ages.

TCD as an independent surveillance signal. Applying the Serfling harmonic regression model independently to the TCD weekly series (Supplementary Figure S3, Supplementary Table S3) yielded 52 epidemic weeks (10.0%) with a cumulative excess of 65,905 TCD-SARI discharges, compared with 50 epidemic weeks and 77,352 excess BCD discharges. The two definitions agreed on the classification of 96.0% (48 of 50) of BCD-derived epidemic weeks (Supplementary Figure S3, panel B). Only 2 BCD epidemic weeks were not also identified by TCD, and 4 TCD epidemic weeks were not also identified by BCD, indicating that the LOS ≤ 7 day filter retains nearly all the epidemic signal of the broader BCD definition while excluding ~22% of cases with prolonged hospitalization. The TCD baseline is essentially a scaled-down version of the BCD baseline, with similar timing of peaks but lower absolute levels.

LOS sensitivity. With LOS ≤ 5 days, 61.4% of BCD cases met the stricter TCD; with LOS ≤ 10 days, 87.8% were captured. The choice of 7 days is therefore neither too tight (sub-50% capture would suggest the cut-off excludes most legitimate community cases) nor too loose (>90% capture would suggest the filter is uninformative). The current cut-off is the appropriate operating point for the LOS distribution observed in Ecuador (Supplementary Table S3).

## 4 Discussion

Three findings structure the interpretation of this analysis. First, hospital discharge data coded using ICD-10 were able to reconstruct coherent national SARI dynamics over a decade, despite the absence of continuous virological surveillance (22, 23). This supports the idea that administrative datasets, when consistently collected, can function as stable indicators of severe respiratory disease burden (24, 25). However, their interpretability depends on explicit case definitions and local validation, as multiple evaluations have shown substantial heterogeneity in sensitivity across pathogens and age groups (26, 27). Second, the apparent reduction of infant SARI after 2014 was not epidemiological. It reflected a shift in coding practices from respiratory J-codes to neonatal P-codes, effectively displacing a high-risk population outside standard surveillance definitions (28). Third, the COVID-19 period was characterized by a decoupling between hospitalization and mortality, with fewer admissions but higher in-hospital case fatality among older adults, suggesting a shift in admission thresholds and case mix under system strain (29).

The U-shape reflects three distinct mechanisms operating across the lifespan. In infants under 1 year, the developing immune system shows a distinctly different, not simply 'weaker', response profile, including suppressed inflammatory responses optimized for the transition from the sterile uterine environment but with reduced capacity to control rapidly replicating respiratory pathogens (30). Combined with smaller airway caliber, lower respiratory reserve, and (in our composite SARI population) the inclusion of premature neonates with surfactant deficiency or congenital aspiration syndromes, this profile explains both the elevated case fatality (4.26%) and the prolonged length of stay (mean 8.8 days). Between approximately 1 and 19 years, immunological maturation is largely complete, comorbid conditions are uncommon, and respiratory reserve is at its life-cycle peak; SARI hospitalizations in this group reflect predominantly self-limited viral and bacterial community-acquired infections, accounting for the very low CFR (0.46–0.70%). The intermediate rise in working-age adults (6.08%) coincides with the accumulation of chronic respiratory and metabolic comorbidities, chronic obstructive pulmonary disease, asthma, diabetes, obesity, that compromise respiratory mechanics and innate antimicrobial function.

Placed within the existing literature, these findings align with, but also extend, current evidence on ICD-based surveillance. Systematic reviews have consistently shown that administrative datasets can track temporal patterns of influenza and SARS-CoV-2 with reasonable concordance when compared with laboratory-confirmed surveillance, although performance is less consistent for RSV and in younger populations (26, 31). Importantly, validation studies have emphasized that coding systems should not be treated as static measurement tools. Instead, they are dynamic constructs shaped by clinical practice, institutional norms, and administrative processes (31). This becomes particularly evident when examining regional variability in respiratory coding, where differences cannot always be explained by disease incidence alone but reflect diagnostic ambiguity and coding behavior. The neonatal coding shift identified in this study represents an extreme manifestation of this phenomenon, but not an isolated one (32).

The temporal disruption observed during 2020–2021 follows a pattern described across multiple countries (33). Non-pharmaceutical interventions implemented during the early phases of the COVID-19 pandemic led to a marked reduction in the circulation of seasonal respiratory viruses, including influenza and RSV (34). Subsequent relaxation of these measures was associated with atypical resurgence patterns, characterized by delayed peaks and extended epidemic periods (35). These dynamics challenge the assumption of stable seasonal baselines and complicate the interpretation of excess hospitalization estimates (36, 37). In this context, deviations from expected patterns should not be interpreted solely as changes in viral transmission but also as reflections of altered healthcare utilization and access (38, 39).

The increase in in-hospital case fatality among older adults during the pandemic likely reflects a combination of biological vulnerability and system-level constraints (40). Age-related immune dysregulation and a higher prevalence of chronic comorbidities contribute to more severe disease (41). At the same time, evidence from hospital-based studies indicates that survival outcomes during COVID-19 waves were influenced by caseload pressure and variability in care delivery (42). When healthcare systems are overwhelmed, admission criteria tend to become more restrictive, and hospitalized cohorts become enriched with critically ill patients. Under such conditions, case fatality ratios may increase even in the absence of changes in pathogen virulence, reflecting a shift in the composition of the treated population rather than a uniform increase in disease severity (43).

A central methodological implication of this study concerns the interpretation of infant respiratory disease burden. Restricting case definitions to standard respiratory ICD codes can lead to substantial underestimation in early life, particularly when neonatal conditions are coded outside conventional respiratory categories (44). Evidence from validation studies indicates that ICD-based algorithms often underestimate disease burden when narrow definitions are used, especially for RSV (32). In this analysis, incorporating neonatal respiratory codes resulted in a marked increase in identified cases, highlighting the magnitude of the distortion (17). This finding indicates a broader issue: surveillance systems are only as accurate as the coding frameworks they rely on. Without periodic auditing and age-specific validation, important segments of the population may remain systematically underrepresented (27).

The strengths of this study lie in its national scope and extended time series, which allow for the identification of structural patterns not observable in smaller or shorter datasets (22). However, several limitations should be considered. The analysis was restricted to principal discharge diagnoses, limiting the ability to account for comorbidities or multiple concurrent conditions (26). Etiological attribution was not possible due to the absence of laboratory data, and this limitation was addressed by explicitly interpreting findings at the syndromic level rather than inferring pathogen-specific dynamics. Coding practices were not directly measured but inferred from temporal trends, and although the use of a composite definition partially mitigates underestimation in infants, residual misclassification cannot be excluded (17, 45). Finally, spatial analyses are ecological and should not be interpreted as causal relationships at the individual level (46, 47). Provincial differences likely reflect a combination of demographic, healthcare, and administrative factors rather than a single underlying mechanism (48).

The Serfling harmonic regression model was developed for temperate-climate influenza surveillance, in which respiratory disease follows a single dominant annual peak. Ecuador's equatorial location produces a bimodal pattern with respiratory peaks associated with the dry and rainy seasons in coastal and Andean regions; our two-harmonic specification (K = 2) is designed to capture this pattern but formal validation in tropical settings remains limited. In addition, OLS Serfling assumes independent residuals; in our data the residual Durbin–Watson statistic was 0.21, indicating strong positive autocorrelation that anti-conservatively widens the count of weeks classified as epidemic. Negative binomial or quasi-Poisson generalized linear models with seasonal and trend terms would be more appropriate for count time series with autocorrelation (11), and we recommend their adoption in future routine SARI surveillance from administrative data in tropical and subtropical contexts. The substantial reduction in cumulative excess from 153,371 in preliminary analyses on a broader 742-week superset to 77,352 in the canonical 522-week 2014–2023 window (clean 522-week window, corrected in this revision) also illustrates the importance of strict adherence to the declared analytic window when interpreting Serfling-derived burden estimates.

From a clinical perspective, these findings emphasize the need for cautious interpretation of hospital-based severity indicators (49). Increases in case fatality may reflect shifts in admission practices rather than intrinsic changes in disease severity (43). This distinction becomes particularly relevant during periods of system stress, when access to care is constrained and only the most severe cases are hospitalized (50). Additionally, underestimation of infant respiratory disease due to coding practices may influence clinical prioritization and resource allocation in neonatal care settings (51, 52).

At the population level, the implications are more consequential, but they require an important qualification. Accurate measurement of hospitalization burden is essential for informing prevention strategies, particularly in the context of emerging RSV interventions (53). Clinical trials and real-world studies have demonstrated the effectiveness of long-acting monoclonal antibodies such as nirsevimab and maternal RSV vaccines in reducing severe disease in infants (54). However, economic evaluations of these interventions are highly sensitive to which component of the infant respiratory burden the intervention is expected to prevent, and the composite SARI definition we use bundles together pathophysiological distinct conditions. When the composite definition is decomposed into its component ICD-10 codes (Supplementary Table S6), only 23,887 of the 114,619 infant SARI discharges identified over 2014–2023 (~21%) carry diagnostic codes that are unambiguously infectious in origin: classic J-codes (J09–J22, including viral pneumonia, bronchiolitis and acute lower respiratory infections) and P23 codes (congenital pneumonia). The remaining 79% carry P22 codes (respiratory distress of the newborn, predominantly surfactant deficiency and transient tachypnoea of prematurity) or P24 codes (neonatal aspiration syndromes, predominantly meconium aspiration), neither of which is an infectious condition and neither of which is preventable by anti-RSV interventions. The full +893% relative increase from the classic J-only definition to the composite definition therefore cannot be interpreted as previously hidden RSV burden; rather, ~11% of that increase reflects infectious neonatal pneumonia (P23, plausibly including RSV but also bacterial and other viral etiologies) and ~89% reflects non-infectious developmental and mechanical conditions of the newborn that are essential to count if the goal is to describe the full burden of severe infant respiratory hospitalization, but that should not enter the denominator of RSV-prevention cost-effectiveness analyses. We therefore recommend that future RSV economic evaluations using Ecuadorian administrative data adopt the J + P23 subset rather than the full composite, and that surveillance studies report the two definitions side-by-side to avoid pathophysiological confounding.

Geographic heterogeneity adds further complexity to the interpretation of these findings. Ecuador's diverse topography, particularly its altitudinal gradients, may influence both disease severity and healthcare utilization (55). Populations living at higher elevations experience chronic hypobaric hypoxia and physiological adaptations that can modify respiratory function. Evidence from pediatric studies suggests that pneumonia incidence and mortality may vary with altitude, although these relationships are influenced by multiple interacting factors, including access to care (56). For this reason, spatial patterns observed in administrative data should be interpreted within their environmental and healthcare context (57).

The reframing has two implications. First, it confirms that the previously described concentration of burden in Pichincha and Guayas is largely demographic, not epidemiological. Second, it directs attention to the Amazonian provinces (Morona Santiago, Pastaza, Zamora Chinchipe), where per-capita excess is two to four times higher than the national average, as priority targets for surveillance strengthening and prevention interventions. Whether this reflects genuinely elevated incidence, lower thresholds for hospital admission, limited outpatient capacity, or differences in coding and case-finding remains a question for primary surveillance studies.

Future research should prioritize the integration of administrative and laboratory data to improve the validity of surveillance systems. Linking discharge records with pathogen-specific testing would allow for local calibration of case definitions and more accurate attribution of disease burden (23). Comparative evaluation of statistical modeling approaches is also warranted, particularly in settings where seasonal patterns are unstable or have been disrupted by external shocks such as the COVID-19 pandemic. Finally, coding practices themselves should be monitored as part of routine surveillance, recognizing that changes in diagnostic behavior can have substantial epidemiological consequences (17).

In conclusion, ICD-10–based hospital discharge data provide a valuable platform for monitoring severe respiratory disease at the population level, particularly in settings with limited laboratory surveillance. However, these data are not neutral representations of disease burden: they are shaped by coding practices, healthcare access, and system-level dynamics, as the neonatal coding shift and the pandemic-related disruption documented in this study illustrate. Building on these findings and on Ecuador's existing surveillance infrastructure, we propose a concrete five-component hybrid model that combines the universal coverage of the INEC administrative registry with targeted virological confirmation through an enhanced sentinel network.

Component 1: Sentinel network footprint. We propose strengthening between eight and ten existing tertiary-care hospitals as ICD-10 + virological sentinels, distributed across Ecuador's four ecoregions to capture climatic and demographic heterogeneity: two coastal sites (one in Guayaquil, one in Manta), four Andean sites (Quito, Cuenca, Ambato, Loja), two Amazonian sites (Tena and Macas, to capture the high per-capita-excess Amazonian pattern identified in 3.9), and one insular site (Galápagos). This footprint extends the current Ecuadorian SIVE-Alerta sentinel system (7), which concentrates virological capacity in Quito and Guayaquil, to provinces such as Morona Santiago and Pastaza, where this study identified the highest per-capita SARI excess (>1,000 per 100,000 inhabitants over 10 years) without virological characterization.

Component 2: Priority pathogens for multiplex PCR confirmation. Each sentinel hospital should perform multiplex RT-PCR (or equivalent platform) on a defined minimum sample of SARI admissions per week, targeting at minimum: influenza A/B (with subtype/lineage discrimination), SARS-CoV-2, respiratory syncytial virus (A and B), human metapneumovirus, parainfluenza viruses 1–4, adenovirus, and rhinovirus/enterovirus. This pathogen list aligns with the WHO Global Influenza Surveillance and Response System (GISRS) extension recommendations and with the Latin American Pediatric Infectious Diseases Society (SLIPE) regional expert consensus on RSV surveillance (4). Specimens should be cross-linked to discharge records by a stable patient identifier to enable case-level integration of administrative and virological data.

Component 3: Routine audit of ICD-10 coding behavior. The coding shift identified in this study (2.7, Supplementary Figure S1) demonstrates that coding behavior is a dynamic exposure with surveillance consequences. We recommend that INEC and the Ministry of Public Health jointly conduct an annual audit of paired J-code (Chapter X) and P-code (Chapter XVI) usage in infants < 1 year, with a triggered review when the year-on-year change in the P-share statistic exceeds ±0.10 (a threshold satisfied only by the 2014 → 2015 transition over our 18-year window). A complementary annual audit of secondary-diagnosis completeness would, if implemented, allow Ecuador to operationalize the Buda et al. (13) Specific Case Definition (SCD) which is currently inaccessible in our data.

Component 4: Coder training and feedback loops. Practical ICD-10 refresher training for hospital coders, with particular attention to the neonatal-respiratory chapter boundary (when to assign a J-code vs. a P-code for an infant respiratory admission), the J18 / U07 boundary during periods of COVID-19 co-circulation, and the BCD/TCD distinction implied by length of stay, should be incorporated into the Ministry's continuing professional development calendar at least biennially. Feedback to coders from automated administrative-data dashboards (e.g., quarterly reports on each hospital's J-vs.-P infant ratio) would provide near-real-time correction for the kinds of systemic shifts documented here.

Component 5: Public-facing administrative dashboards with virological denominators. Building on the existing INEC open-data infrastructure and on the lessons learned during the COVID-19 pandemic (7), we propose quarterly public publication of administrative-data-derived SARI dashboards at provincial granularity, with virological denominators from the sentinel network to translate ICD-10 SARI counts into pathogen-attributed estimates. The dashboards should include the headline weekly Serfling baseline and 95% prediction interval, age-stratified counts under both the BCD and the J + P23 infant-infectious subset (2.8, Supplementary Table S6), and a coding-stability indicator (the J-vs-P infant ratio described in Component 3) as a quality-control flag for end users.

Operationalization horizon: A pragmatic phasing would begin with Components 3 and 4 (coding audits and coder training, implementable within 12 months and at minimal incremental cost), proceed in parallel with Component 5 (dashboard development, 12–24 months), and culminate in the laboratory-network expansion of Components 1 and 2 (24–48 months, subject to multiplex-PCR capacity-building). This sequencing privileges immediate strengthening of the administrative data already available, which, as this study demonstrates, can support a substantial proportion of national SARI surveillance objectives at near-zero marginal cost, while building the laboratory infrastructure required for etiological attribution at a feasible pace for a middle-income setting.

In summary, Ecuador is well-positioned to lead the region in operationalizing the hybrid surveillance model that the WHO and PAHO have recommended for SARI in equatorial Latin America. The findings of this study, the empirical verification of a decade of ICD-10 surveillance, the documentation of a coding shift that distorted standard case definitions, and the identification of Amazonian provinces with disproportionate per-capita excess, provide both the methodological foundation and the operational priorities for the next phase of national SARI surveillance.

## Data Availability

The hospital discharge microdata analysed in this study are publicly available from the Instituto Nacional de Estadística y Censos (INEC) of Ecuador (https://www.ecuadorencifras.gob.ec). The authors are not permitted to redistribute these data, and their reuse is subject to INEC's terms of use. Aggregated data and the analysis code are available from the corresponding author upon reasonable request.

## References

[1] Paulson KR Kamath AM Alam T Bienhoff K Abady GG Abbas J . Global, regional, and national progress towards Sustainable Development Goal 3.2 for neonatal and child health: all-cause and cause-specific mortality findings from the Global Burden of Disease Study 2019. Lancet. (2021) 398:870–905. doi: 10.1016/S0140-6736(21)01207-1 34416195 PMC8429803

[2] Troeger C Blacker B Khalil IA Rao PC Cao J Zimsen SRM . Estimates of the global, regional, and national morbidity, mortality, and aetiologies of lower respiratory infections in 195 countries, 1990–2016: a systematic analysis for the Global Burden of Disease Study 2016. Lancet Infect Dis. (2018) 18:1191–210. doi: 10.1016/S1473-3099(18)30310-4 30243584 PMC6202443

[3] Ciapponi A Palermo MC Sandoval MM Baumeister E Ruvinsky S Ulloa-Gutierrez R . Respiratory syncytial virus disease burden in children and adults from Latin America: a systematic review and meta-analysis. Front Public Health. (2024) 12:1377968. doi: 10.3389/fpubh.2024.1377968 39478747 PMC11521816

[4] Debbag R Ávila-Agüero ML Brea J Brenes-Chacon H Colomé M de Antonio R . Confronting the challenge: a regional perspective by the Latin American Pediatric Infectious Diseases Society (SLIPE) expert group on respiratory syncytial virus—tackling the burden of disease and implementing preventive solutions. Front Pediatr. (2024) 12:1386082. doi: 10.3389/fped.2024.1386082 39144471 PMC11322482

[5] Castro C Leiva V Giampaoli V Prieto D Martin-Barreiro C Viana J. Asthma and severe acute respiratory infections: a stratified analysis of mortality patterns in Brazil. BMC Public Health. (2025) 25:3168. doi: 10.1186/s12889-025-24264-0 41029327 PMC12487218

[6] Radin JM Katz MA Tempia S Talla Nzussouo N Davis R Duque J . Influenza Surveillance in 15 Countries in Africa, 2006–2010. J Infect Dis. (2012) 206:S14–21. doi: 10.1093/infdis/jis606 23169960

[7] Gualotuña-Suntaxi J Pérez-Muñoz D Zambrano-Villacres R Zambrano AK Simancas-Racines D Angamarca-Iguago J. Available evidence on integrating COVID-19 into sentinel surveillance systems: a scoping review. Medwave. (2025). doi: 10.5867/medwave.2025.09.3026 41115313

[8] Douce RW Aleman W Chicaiza-Ayala W Madrid C Sovero M Delgado F . Sentinel surveillance of influenza-like-illness in two cities of the tropical country of Ecuador: 2006–2010. PLoS ONE. (2011) 6:e22206. doi: 10.1371/journal.pone.0022206 21887216 PMC3160842

[9] Nair H Nokes DJ Gessner BD Dherani M Madhi SA Singleton RJ . Global burden of acute lower respiratory infections due to respiratory syncytial virus in young children: a systematic review and meta-analysis. Lancet. (2010) 375:1545–55. doi: 10.1016/S0140-6736(10)60206-1 20399493 PMC2864404

[10] Thompson WW Shay DK Weintraub E Brammer L Cox N Anderson LJ . Mortality Associated With Influenza and Respiratory Syncytial Virus in the United States. JAMA. (2003) 289:179. doi: 10.1001/jama.289.2.179 12517228

[11] Goldstein E Viboud C Charu V Lipsitch M. Improving the Estimation of Influenza-Related Mortality Over a Seasonal Baseline. Epidemiology. (2012) 23:829–38. doi: 10.1097/EDE.0b013e31826c2dda 22992574 PMC3516362

[12] Nielsen J Mazick A Andrews N Detsis M Fenech TM Flores VM„ et al. Pooling European all-cause mortality: methodology and findings for the seasons 2008/2009 to 2010/2011. Epidemiol Infect. (2013) 141:1996–2010. doi: 10.1017/S0950268812002580 23182146 PMC9151408

[13] Buda S Tolksdorf K Schuler E Kuhlen R Haas W. Establishing an ICD-10 code based SARI-surveillance in Germany – description of the system and first results from five recent influenza seasons. BMC Public Health. (2017) 17:612. doi: 10.1186/s12889-017-4515-1 28666433 PMC5493063

[14] Rodrigues E Machado A Silva S Nunes B. Excess pneumonia and influenza hospitalizations associated with influenza epidemics in Portugal from season 1998/1999 to 2014/2015. Influenza Other Respir Viruses. (2018) 12:153–60. doi: 10.1111/irv.12501 29460423 PMC5818339

[15] Chicaiza-Ayala W Henríquez-Trujillo AR Ortiz-Prado E Douce RW Coral-Almeida M. The burden of acute respiratory infections in Ecuador 2011–2015. PLoS ONE. (2018) 13:e0196650. doi: 10.1371/journal.pone.0196650 29715314 PMC5929540

[16] Instituto Nacional de Estadística y Censos (INEC). Camas y Egresos Hospitalarios — Información Histórica. (2024). Available: https://www.ecuadorencifras.gob.ec/camas-y-egresos-hospitalarios-informacion-historica/ [accessed 25 Feb 2026]

[17] Walkowiak MP Walkowiak D Walkowiak J. Breaking ICD Codes: Identifying Ambiguous Respiratory Infection Codes via Regional Diagnosis Heterogeneity. Ann Fam Med. (2025) 23:9–15. doi: 10.1370/afm.3192 39870533 PMC11772040

[18] Cai W Buda S Schuler E Hirve S Zhang W Haas W. Risk factors for hospitalized respiratory syncytial virus disease and its severe outcomes. Influenza Other Respir Viruses. (2020) 14:658–70. doi: 10.1111/irv.12729 32064773 PMC7578333

[19] Serfling RE. Methods for current statistical analysis of excess pneumonia-influenza deaths. Public Health Rep. (1963) 78:494–506. doi: 10.2307/4591848 19316455 PMC1915276

[20] Instituto Nacional de Estadística y Censos (INEC). Proyecciones poblacionales Ecuador 2010–2050. (2020).

[21] World Health Organization. WHO surveillance case definitions for ILI and SARI. 2014. Available: https://www.who.int/teams/global-influenza-programme/surveillance-and-monitoring [accessed 18 Feb 2026]

[22] Seppälä E Bøås H Dahl J Stålcrantz J Stecher M Tønnessen R . Registry-Based Surveillance of Severe Acute Respiratory Infections in Norway During 2021–2024. Influenza Other Respir Viruses. (2025) 19:e70080. doi: 10.1111/irv.70080 39950571 PMC11826439

[23] Lomholt FK Møller KL Nielsen J Valentiner-Branth P Vestergaard LS. Surveillance of severe acute respiratory infections using ICD-10 diagnosis codes and national electronic health records, Denmark, 2022 to 2024. Eurosurveillance. (2025) 30:2400801. doi: 10.2807/1560-7917.ES.2025.30.33.2400801 40843520 PMC12372893

[24] Glatman-Freedman A Gur-Arie L Dichtiar R Keinan-Boker L Bromberg M. Laboratory-confirmed respiratory syncytial virus (RSV) hospitalizations: a national all ages cross-section evaluation, 2020–2024. Isr J Health Policy Res. (2025) 14:36. doi: 10.1186/s13584-025-00693-5 40500807 PMC12153084

[25] Bayer LJ Brösamle C Brestrich G Najafi B von Eiff C Hösemann C . RSV Monitoring in Germany: A Critical Overview of Available Surveillance Systems. J Clin Med. (2025) 14:7487. doi: 10.3390/jcm14217487 41226883 PMC12608847

[26] Sanchez Ruiz MA Marques DF Lomholt FK Vestergaard LS Monge S Lozano Álvarez M . Surveillance of severe acute respiratory infections associated with SARS-CoV-2, influenza virus and RSV using ICD-10 codes: a case definition accuracy study across five European countries, 2021 to 2023. Eurosurveillance. (2025) 30:2400748. doi: 10.2807/1560-7917.ES.2025.30.27.2400748 40642769 PMC12262114

[27] Agyei-Manu E Atkins N Nundy M St-Jean C Gornall-Wick A Birley E . Characteristics of influenza, SARS-CoV-2, and RSV surveillance systems that utilise ICD-coded data: a systematic review. J Glob Health. (2025) 15:04177. doi: 10.7189/jogh.15.04177 40406976 PMC12100576

[28] Cai W Tolksdorf K Hirve S Schuler E Zhang W Haas W . Evaluation of using ICD-10 code data for respiratory syncytial virus surveillance. Influenza Other Respir Viruses. (2020) 14:630–7. doi: 10.1111/irv.12665 31206246 PMC7578302

[29] Bøås H Seppälä E Veneti L Stålcrantz J Berild JD Dahl J . Changed epidemiology of influenza and RSV hospitalizations after the emergence of SARS-CoV-2 in Norway, 2017–2024. Ann Epidemiol. (2025) 108:77–84. doi: 10.1016/j.annepidem.2025.06.010 40543765

[30] Sedney CJ Harvill ET. The neonatal immune system and respiratory pathogens. Microorganisms. (2023) 11:1597. doi: 10.3390/microorganisms11061597 37375099 PMC10301501

[31] Mira-Iglesias A López-Lacort M Bricout H Loiacono M Carballido-Fernández M Mollar-Maseres J . Accuracy of ICD Influenza Discharge Diagnosis Codes in Hospitalized Adults From the Valencia Region, Spain, in the Pre-COVID-19 Period 2012/2013 to 2017/2018. Influenza Other Respir Viruses. (2025) 19:e70069. doi: 10.1111/irv.70069 39909969 PMC11798732

[32] Hamilton MA Calzavara A Emerson SD Djebli M Sundaram ME Chan AK . Validating International Classification of Disease 10th Revision algorithms for identifying influenza and respiratory syncytial virus hospitalizations. Katzenellenbogen J, editor PLoS One. (2021) 16:e0244746. doi: 10.1371/journal.pone.0244746 33411792 PMC7790248

[33] Chow EJ Uyeki TM Chu HY. The effects of the COVID-19 pandemic on community respiratory virus activity. Nat Rev Microbiol. (2022) 21:195–210. doi: 10.1038/s41579-022-00807-9 36253478 PMC9574826

[34] Jelley L Douglas J O'Neill M Berquist K Claasen A Wang J . Spatial and temporal transmission dynamics of respiratory syncytial virus in New Zealand before and after the COVID-19 pandemic. Nat Commun. (2024) 15:9758. doi: 10.1038/s41467-024-53998-5 39528493 PMC11555088

[35] Heemskerk S Baliatsas C Stelma F Nair H Paget J Spreeuwenberg P. Assessing the Impact of Non-Pharmaceutical Interventions During the COVID-19 Pandemic on RSV Seasonality in Europe. Influenza Other Respir Viruses. (2025) 19:e70066. doi: 10.1111/irv.70066 39838552 PMC11750802

[36] Dewey G Meyer AG Garcia RG Santillana M. Uncovering the post-pandemic timing of influenza, RSV, and COVID-19 driving seasonal influenza-like illness in the United States: a retrospective ecological study. Lancet Reg Health Am. (2026) 55:101359. doi: 10.1016/j.lana.2025.101359 41551297 PMC12804126

[37] Wang H Kwok KO Li R Riley S. Forecasting regional COVID-19 hospitalisation in England using ordinal machine learning method. Epidemics. (2025) 53:100856. doi: 10.1016/j.epidem.2025.100856 41027240

[38] Bayindir EE Schreyögg J. Trust and the impact of state interventions on healthcare utilization during the COVID-19 pandemic in Germany: An instrumental variables approach. Health Policy. (2026) 168:105598. doi: 10.1016/j.healthpol.2026.105598 41802378

[39] Motiei M Hassanzadeh Rad A Badeli H Bayat R. Hospitalization dynamics during COVID-19: Insights into disease trends and patient outcomes. PLoS One. (2025) 20:e0321269. doi: 10.1371/journal.pone.0321269 40294040 PMC12036843

[40] Zinatizadeh MR Zarandi PK Ghiasi M Kooshki H Mohammadi M Amani J . Immunosenescence and inflamm-ageing in COVID-19. Ageing Res Rev. (2023) 84:101818. doi: 10.1016/j.arr.2022.101818 36516928 PMC9741765

[41] Kadri SS Sun J Lawandi A Strich JR Busch LM Keller M . Association Between Caseload Surge and COVID-19 Survival in 558 U.S. Hospitals, March to August 2020. Ann Intern Med. (2021) 174: 1240–1251. doi: 10.7326/M21-1213 34224257 PMC8276718

[42] Ongoli A Opio B Opollo MS Akello AR Kiweewa F Omech B. Mortality and associated factors among hospitalised COVID-19 patients in Lira regional referral hospital, a cross-sectional study. BMJ Open. (2025) 15:e094648. doi: 10.1136/bmjopen-2024-094648 41146385 PMC12557795

[43] Chakraborty C Bhattacharya M Lee S-S. Excess mortality in older adults and cumulative excess mortality across all ages during the COVID-19 pandemic in the 20 countries with the highest mortality rates worldwide. Osong Public Health Res Perspect. (2025) 16:42–58. doi: 10.24171/j.phrp.2024.0186 39967006 PMC11917389

[44] Bierrenbach AL Ranzani OT. Evaluating the accuracy of ICD-10 codes for syncytial respiratory virus diagnosis in hospitalized patients: A record-linkage study (2022–2023). PLoS ONE. (2025) 20:e0319436. doi: 10.1371/journal.pone.0319436 40153396 PMC11952245

[45] Reyburn R Russell FM Munywoki PK Franzel L Huong TTG Feikin D . Designing effectiveness and impact studies for respiratory syncytial virus immunisation in low- and middle-income countries. Vaccine. (2025) 61:127397. doi: 10.1016/j.vaccine.2025.127397 40540780

[46] Nazia N Butt ZA Bedard ML Tang W-C Sehar H Law J. Methods Used in the Spatial and Spatiotemporal Analysis of COVID-19 Epidemiology: A Systematic Review. Int J Environ Res Public Health. (2022) 19:8267. doi: 10.3390/ijerph19148267 35886114 PMC9324591

[47] Tesema GA Tessema ZT Heritier S Stirling RG Earnest A A. Systematic Review of Joint Spatial and Spatiotemporal Models in Health Research. Int J Environ Res Public Health. (2023) 20:5295. doi: 10.3390/ijerph20075295 37047911 PMC10094468

[48] Johnson EK Sylte D Chaves SS Li Y Mahe C Nair H . Hospital utilization rates for influenza and RSV: a novel approach and critical assessment. Popul Health Metr. (2021) 19:31. doi: 10.1186/s12963-021-00252-5 34126993 PMC8204427

[49] Bottle A Faitna P Brett S Aylin P. Factors associated with, and variations in, COVID-19 hospital death rates in England's first two waves: observational study. BMJ Open. (2022) 12:e060251. doi: 10.1136/bmjopen-2021-060251 35772812 PMC9247323

[50] Myers LC Liu VX. The COVID-19 Pandemic Strikes Again and Again and Again. JAMA Netw Open. (2022) 5:e221760. doi: 10.1001/jamanetworkopen.2022.1760 35262720

[51] Gantenberg JR Thompson KD van Aalst R Smith DM Richards M Nelson CB . Inpatient service utilization amongst infants diagnosed with Respiratory Syncytial Virus infection (RSV) in the United States. PLoS ONE. (2025) 20:e0317367. doi: 10.1371/journal.pone.0317367 39804848 PMC11730397

[52] Saha S Saha S Kanon N Hooda Y Islam MS Islam S . Health-care burden related to respiratory syncytial virus in a resource-constrained setting: a prospective observational study. Lancet Glob Health. (2025) 13:e1072–81. doi: 10.1016/S2214-109X(25)00048-8 40412396 PMC12100462

[53] Havers FP Whitaker M Pham H Anglin O Milucky J Patel K . 2209. RSV-associated Hospitalizations in Adults Aged ≥18 Years and the Impact of the COVID-19 Pandemic in the United States, October 2018 – February 2022. Open Forum Infect Dis. (2022) 9:ofac492.182. doi: 10.1093/ofid/ofac492.1828

[54] Venkatesan P. First RSV vaccine approvals. Lancet Microbe. (2023) 4:e632. doi: 10.1016/S2666-5247(23)00195-7 37390835

[55] Hu K Li C Yang X Ou S Zhang X Xiao D . From infectious diseases to chronic diseases: the paradigm shift of spatial epidemiology in disease prevention and control. Front Public Health. (2025) 13:1698964. doi: 10.3389/fpubh.2025.1698964 41164843 PMC12558956

[56] Crocker ME Hossen S Goodman D Simkovich SM Kirby M Thompson LM . Effects of high altitude on respiratory rate and oxygen saturation reference values in healthy infants and children younger than 2 years in four countries: a cross-sectional study. Lancet Glob Health. (2020) 8:e362–73. doi: 10.1016/S2214-109X(19)30543-1 32087173 PMC7034060

[57] Cortes-Ramirez J Wilches-Vega JD Michael RN Singh V Paris-Pineda OM. Estimating spatial disease rates using health statistics without geographic identifiers. GeoJournal. (2023) 88:4573–83. doi: 10.1007/s10708-022-10822-1

