# Supplementary material for: Reconstructing severe acute respiratory infection dynamics from ICD-10 hospital discharge data: a 10-year analysis of 11.2 million discharges in Ecuador, 2014–2023
Source: Front Public Health. 2026 Jul 22;14:1863826. doi: 10.3389/fpubh.2026.1863826 (PMC13438157; doi:10.3389/fpubh.2026.1863826)
Supplement: Supplementary file 1 [file Supplementary_File_1.docx]

Supplementary Material

## Reconstructing Severe Acute Respiratory Infection Dynamics from ICD-10 Hospital Discharge Data: A 10-Year Analysis of 11.2 Million Discharges in Ecuador, 2014–2023

# S1 Empirical verification of the ICD-10 coding shift

## S1.1 Methods

An abrupt change in infant J-code usage appears at the very start of the 2014–2023 study period. To test whether this reflects a systemic shift in coding practice rather than a localized registration artefact, we extended the analysis seven years backward (2007–2013) and one year forward (2024). We used the same INEC Egresos Hospitalarios microdata files, available from the public open-data portal. The enlarged dataset comprised 19,859,700 discharge records spanning 2007–2024 (1,059,773 infants aged <1 year).

We extracted all discharges with a three-character ICD-10 prefix in J09–J22 (respiratory chapter) or P22–P24 (perinatal chapter) and a recorded age below one year that is, infants recorded as aged in days or months, or aged in completed years with age equal to zero. For each year and each of the 24 provinces, we computed a P-share statistic, defined as P / (J + P) the fraction of cases coded under the perinatal chapter.

The `causa3` field has reliability problems (missing values in 2011 and free-text descriptors in 2024). To avoid these, we derived the three-character ICD-10 prefix from `cau_cie10` the four-character code, which is more uniformly populated by taking its first three characters (one letter followed by two digits, e.g. J18).

## S1.2 Figure S1 National and provincial decomposition of the J→P shift

**Figure S1.** Empirical decomposition of the ICD-10 coding shift in infants under 1 year, Ecuador 2007–2024.

**Panel A:** National stacked bar chart showing yearly counts of infant respiratory discharges coded under the respiratory chapter (J09–J22, blue) versus the perinatal chapter (P22–P24, red). The vertical dashed line marks the 2014→2015 transition. Both code families coexist during 2007–2014 (national P-share 0.38–0.55); in 2015 J-coding collapses to ~200 cases nationwide while P-coding remains stable; in 2024 (outside the study period) a partial reversal returns J-codes to 7,580 cases.

**Panel B:** Heatmap of provincial P-share (P / (P+J)) by year and province. Blue cells = J-dominant; red cells = P-dominant. The synchronized red shift in 2015 across 23 of 24 provinces (95.8% with ≥90% drop in J-coding) demonstrates that the transition was systemic rather than hospital-specific. Galápagos (small population) is the only province where the pattern is noisy.

## S1.3 Table S1a National infant respiratory coding by year (2007–2024)

| **Year** | **Infants <1y** | **J09–J22** | **P22–P24** | **P-share** |
| --- | --- | --- | --- | --- |
| 2007 | 54,477 | 9,591 | 5,981 | 0.384 |
| 2008 | 65,647 | 9,882 | 9,007 | 0.477 |
| 2009 | 69,938 | 10,695 | 9,283 | 0.465 |
| 2010 | 77,926 | 12,032 | 8,902 | 0.425 |
| 2011 | 73,653 | 10,710 | 9,658 | 0.474 |
| 2012 | 79,354 | 13,333 | 11,370 | 0.460 |
| 2013 | 77,141 | 11,792 | 10,887 | 0.480 |
| 2014 | 82,792 | 10,606 | 12,821 | 0.547 |
| **2015** | **50,787** | **197** | **10,725** | **0.982** ← |
| 2016 | 50,320 | 215 | 10,647 | 0.980 |
| 2017 | 51,201 | 227 | 10,681 | 0.979 |
| 2018 | 50,806 | 189 | 10,172 | 0.982 |
| 2019 | 51,294 | 250 | 9,319 | 0.974 |
| 2020 | 43,893 | 113 | 8,005 | 0.986 |
| 2021 | 45,433 | 74 | 9,609 | 0.992 |
| 2022 | 48,535 | 204 | 10,362 | 0.981 |
| 2023 | 48,278 | 186 | 10,017 | 0.982 |
| 2024 | 38,298 | 7,580 | 3,013 | 0.284 |

The 2014→2015 jump in P-share (+0.435, the largest in the 18-year series) coincides with a 98.1% collapse in J-coding nationwide. The 2024 partial reversal (J-coding rebounds to 7,580) occurs outside the study period and reinforces the interpretation that the 2014–2015 transition reflects administrative behavior rather than disease incidence.

## S1.4 Table S1b Provincial × year P-share matrix

The complete provincial × year P-share matrix comprises all 24 provinces across the full 18-year series (2007–2024). For clarity, a representative subset of provinces is presented below to illustrate the magnitude and synchrony of the 2014→2015 transition:

| **Province** | **P-share 2014** | **P-share 2015** | **Δ** | **J-count drop ≥90%?** |
| --- | --- | --- | --- | --- |
| Pichincha | 0.51 | 0.99 | +0.48 | Yes |
| Guayas | 0.58 | 0.98 | +0.40 | Yes |
| Manabí | 0.47 | 0.97 | +0.50 | Yes |
| Azuay | 0.52 | 0.98 | +0.46 | Yes |
| Morona Santiago | 0.62 | 0.99 | +0.37 | Yes |
| Galápagos | 0.71 | 0.93 | +0.22 | (small n) |

**Provincial synchrony summary:** 23 of 24 provinces (95.8%) showed J-count drop ≥90% between 2014 and 2015. The exception (Galápagos) has population <40,000 and annual infant respiratory admission counts in single digits.

# S2 — Serfling sensitivity analysis

## S2.1 Rationale

An earlier version of this analysis reported 742 epidemiological weeks without explicit treatment of the 2014 transition year. On re-examination, we identified that this fit had inadvertently included 220 weeks with admission dates outside the declared 2014–2023 window (140 weeks from 2010–2013 and 80 weeks from implausible or corrupted dates such as 1959, 1987, and 2004). Restricting the analysis to the canonical 522-week window yields substantively different absolute numbers; we therefore present four sensitivity scenarios:

- **S0** — v1 replication (full 742-week superset, with leakage). Reported for transparency only.

- **S1** — Main analysis (clean 522 weeks, 2014–2023).

- **S2** — Exclude the 2014 transition year (470 weeks) from both fit and excess attribution.

- **S3** — Pre-pandemic fit only (2014–2019, 313 weeks) with out-of-sample prediction for 2020–2023 (Heemskerk 2025 approach for COVID-disrupted series).

## S2.2 Figure S2 Three-panel sensitivity comparison

**Figure S2.** Serfling sensitivity analysis under three fitting strategies. Each panel shows observed weekly BCD-SARI counts (grey), fitted Serfling baseline (blue), upper 95% threshold (dashed blue), and epidemic weeks (red dots). Cumulative excess and Durbin–Watson diagnostic shown in inset.

## S2.3 Table S2 — Numerical comparison

| **Scenario** | **n weeks** | **Epi weeks** | **Epi %** | **Cumulative excess** | **95% PI** | **Durbin–Watson** |
| --- | --- | --- | --- | --- | --- | --- |
| **S0 v1 replication (with leakage)** | 742 | 69 | 9.3% | **153,371** | — | 0.08 |
| **S1 main (clean 2014–2023)** | **522** | **50** | **9.6%** | **77,352** | **70,030–85,982** | **0.21** |
| S2 exclude 2014 | 470 | 42 | 8.9% | 72,246 | 64,917–81,061 | 0.22 |
| S3 pre-pandemic fit + OOS | 313 fit / 522 total | 97 | 18.6% | 40,957 | 36,618–46,554 | 1.19 |

**Notes:**

- S0 confirms exact reproduction of the v1 reported number (153,371). The discrepancy with S1 is entirely attributable to the 220-week pre-2014 leakage.

- Excluding 2014 (S2) changes cumulative excess by only −6.6% relative to S1, indicating that the coding-transition year contributes a small fraction of the headline excess.

- S3 yields the most conservative excess estimate (40,957), reflecting a baseline fitted exclusively on pre-pandemic data and projected out-of-sample through the COVID-19 disruption.

- All scenarios show strong residual positive autocorrelation (DW far from 2.0), indicating that the OLS-based 95% prediction interval is anti-conservative. Negative binomial or quasi-Poisson GLMs would be more appropriate for future routine surveillance.

# S3 — TCD deep-dive and length-of-stay sensitivity

## S3.1 Figure S3 — BCD vs TCD comparison

**Figure S3. (A)** National BCD vs TCD weekly time series with independent Serfling baselines and 95% thresholds. **(B)** Overlap of epidemic-week classifications: of 50 BCD-derived epidemic weeks, 48 (96.0%) are also flagged by TCD; only 2 are BCD-only and 4 are TCD-only.

## S3.2 Table S3 LOS sensitivity for the TCD definition

| **LOS threshold** | **n meeting TCD** | **% of BCD** |
| --- | --- | --- |
| ≤ 5 days | 333,344 | 61.45% |
| **≤ 7 days (main analysis)** | **420,371** | **77.49%** |
| ≤ 10 days | 476,079 | 87.76% |

Based on composite BCD pool (J09–J22 plus P22–P24 for infants <1 year), 2014–2023, n = 542,457 records with valid LOS. Median LOS = 5 days, mean = 6.05 days, IQR 3–7 days. The TCD definition retained in the main analysis (≤ 7 days) corresponds to approximately the third quartile of the empirical LOS distribution, supporting it as a defensible separation between rapidly-resolving (community-acquired) and slowly-resolving (nosocomial, complicated, or chronic) respiratory hospitalisations.

## S3.3 BCD/TCD ratio by age (clean 522-week window)

| **Age group** | **Total BCD** | **Total TCD** | **TCD/BCD** |
| --- | --- | --- | --- |
| <1 year | 108,849 | 71,514 | 65.7% |
| 1–4 years | 175,768 | 152,798 | 86.9% |
| 5–19 years | 97,300 | 83,336 | 85.6% |
| 20–64 years | 56,302 | 41,744 | 74.1% |
| ≥65 years | 99,419 | 68,097 | 68.5% |

# S4 Provincial SARI: full 24-province summary (clean 522-week window)

## S4.1 Table S4

Full Serfling and population-adjusted summary for all 24 provinces, 2014–2023. Population denominators are INEC 2018 projections.

| **#** | **Province** | **Total BCD** | **Epi wks** | **Epi %** | **Cum. excess** | **Mean wkly** | **Peak wkly** | **Deaths** | **CFR %** | **Pop 2018** | **Rate /100k/yr** | **Excess /100k** |
| --- | --- | --- | --- | --- | --- | --- | --- | --- | --- | --- | --- | --- |
| 1 | Azuay | 38,664 | 80 | 15.3 | 7,168 | 74.1 | 178 | 1,894 | 4.90 | 870,000 | 444.4 | 824.0 |
| 2 | Bolívar | 4,898 | 102 | 19.5 | 1,408 | 9.4 | 33 | 131 | 2.67 | 208,000 | 235.5 | 677.4 |
| 3 | Cañar | 12,510 | 75 | 14.4 | 2,810 | 24.0 | 74 | 371 | 2.97 | 280,000 | 446.8 | 1,003.9 |
| 4 | Carchi | 6,205 | 79 | 15.1 | 1,520 | 11.9 | 38 | 239 | 3.85 | 182,000 | 340.9 | 835.3 |
| 5 | Cotopaxi | 11,532 | 86 | 16.5 | 2,794 | 22.1 | 72 | 423 | 3.67 | 480,000 | 240.2 | 582.1 |
| 6 | Chimborazo | 18,976 | 81 | 15.5 | 3,979 | 36.4 | 99 | 709 | 3.74 | 497,000 | 381.8 | 800.7 |
| 7 | El Oro | 28,361 | 61 | 11.7 | 4,500 | 54.3 | 126 | 1,437 | 5.07 | 715,000 | 396.7 | 629.4 |
| 8 | Esmeraldas | 11,910 | 95 | 18.2 | 2,261 | 22.8 | 55 | 615 | 5.16 | 632,000 | 188.4 | 357.8 |
| 9 | Guayas | 111,877 | 82 | 15.7 | 15,366 | 214.3 | 518 | 6,221 | 5.56 | 4,350,000 | 257.2 | 353.3 |
| 10 | Imbabura | 15,809 | 97 | 18.6 | 3,836 | 30.3 | 82 | 379 | 2.40 | 467,000 | 338.5 | 821.5 |
| 11 | Loja | 19,712 | 74 | 14.2 | 3,844 | 37.8 | 135 | 685 | 3.48 | 516,000 | 382.0 | 745.1 |
| 12 | Los Ríos | 18,834 | 73 | 14.0 | 3,578 | 36.1 | 96 | 569 | 3.02 | 884,000 | 213.1 | 404.8 |
| 13 | Manabí | 37,296 | 74 | 14.2 | 6,576 | 71.4 | 158 | 1,477 | 3.96 | 1,517,000 | 245.9 | 433.5 |
| 14 | **Morona Santiago** | 15,103 | **98** | **18.8** | 3,054 | 28.9 | 68 | 282 | **1.87** | 198,000 | **762.8** | **1,542.7** |
| 15 | Napo | 4,234 | 89 | 17.0 | 1,125 | 8.1 | 26 | 88 | 2.08 | 130,000 | 325.7 | 865.4 |
| 16 | Pastaza | 4,202 | 84 | 16.1 | 1,154 | 8.0 | 35 | 94 | 2.24 | 106,000 | 396.4 | 1,089.1 |
| 17 | Pichincha | 119,765 | 97 | 18.6 | 29,782 | 229.4 | 748 | 3,733 | 3.12 | 3,050,000 | 392.7 | 976.5 |
| 18 | Tungurahua | 20,593 | 100 | 19.2 | 5,630 | 39.5 | 127 | 837 | 4.06 | 565,000 | 364.5 | 996.6 |
| 19 | Zamora Chinchipe | 4,152 | 93 | 17.8 | 1,237 | 8.0 | 31 | 97 | 2.34 | 115,000 | 361.0 | 1,076.1 |
| 20 | Galápagos | 581 | 90 | 17.2 | 326 | 1.1 | 7 | 16 | 2.75 | 32,000 | 181.6 | 1,019.9 |
| 21 | Sucumbíos | 6,100 | 79 | 15.1 | 1,259 | 11.7 | 34 | 279 | 4.57 | 230,000 | 265.2 | 547.8 |
| 22 | Orellana | 3,187 | 95 | 18.2 | 924 | 6.1 | 20 | 61 | 1.91 | 163,000 | 195.5 | 567.3 |
| 23 | Santo Domingo | 15,304 | 80 | 15.3 | 2,845 | 29.3 | 75 | 564 | 3.69 | 454,000 | 337.1 | 626.8 |
| 24 | Santa Elena | 7,833 | 85 | 16.3 | 1,846 | 15.0 | 40 | 205 | 2.62 | 404,000 | 193.9 | 457.0 |

Highlighted cells (Morona Santiago) identify the high-rate / low-CFR Amazonian archetype with the highest population-adjusted excess in the country (1,542.7 per 100,000), despite contributing only 3,054 cases in absolute terms.

# S5 — Population denominators

INEC 2018 population projections (midpoint of the 2014–2023 study period) were used as the denominator for all per-100,000 rate calculations in Table S4 and in the revised Results §3.9. Alternative denominators (mean of 2014–2023 projections, or 2020 census) yielded essentially identical results (<2% relative difference) and are not reported separately.

The Rate /100k/yr column is computed as: (total BCD across 10 years × 100,000) / (population × 10).

The Excess /100k column is computed as: (cumulative 10-year Serfling excess × 100,000) / population.

# S6 — Decomposition of the composite infant SARI definition

## S6.1 Rationale

The composite infant SARI definition introduced in §2.6 of the main text (J09–J22 plus P22–P24 in infants <1 year) bundles pathophysiologically distinct conditions. Because the implication of the +893% relative increase from the classic J-only definition to the composite for RSV-prevention cost-effectiveness modelling depends on the proportion of the composite that is *infectious*, we report here the decomposition by sub-code.

## S6.2 Table S6 — ICD-10 code-level decomposition of composite infant SARI (2014–2023)

| **Code** | **Description** | **n 2014–2023** | **Nature** |
| --- | --- | --- | --- |
| J09–J22 (classic) | Influenza, viral & bacterial pneumonia, bronchiolitis, bronchitis, unspecified ALRI | **12,261** | **Infectious** |
| P22 | Respiratory distress of newborn (surfactant deficiency, RDS of prematurity, transient tachypnoea of the newborn) | **84,722** | **Non-infectious** (developmental) |
| P23 | Congenital pneumonia | **11,626** | **Infectious** (RSV-plausible) |
| P24 | Neonatal aspiration syndromes (predominantly meconium aspiration) | **6,010** | **Non-infectious** (mechanical) |
| Composite (J + P22 + P23 + P24) | Main analysis definition | **114,619** | Mixed |
| **Composite (J + P23 only)** | **Restricted sensitivity definition for RSV/infectious modelling** | **23,887** | **Infectious only** |

## S6.3 Interpretation

Of the +893% relative increase from the classic J-only definition to the full composite, only 11% corresponds to P23 (plausibly infectious neonatal pneumonia); the remaining 89% corresponds to P22 + P24 (non-infectious neonatal respiratory conditions). The full composite is retained as the main definition because it captures the *total burden of severe infant respiratory hospitalization* — relevant for NICU capacity planning, surfactant therapy demand, and mechanical ventilation logistics — but the J + P23 restricted subset should be used as the appropriate denominator for RSV-prevention cost-effectiveness analyses (see main-text Discussion paragraph on RSV/nirsevimab).

# Data availability

All analytical scripts and intermediate outputs supporting this revision are archived locally and are available from the corresponding author on request. The primary microdata are available from the INEC open-data portal (https://www.ecuadorencifras.gob.ec/camas-y-egresos-hospitalarios/).

*End of Supplementary Material.*
